# 3D Cellular Architecture Affects MicroRNA and Protein Cargo of Extracellular Vesicles

**DOI:** 10.1002/advs.201800948

**Published:** 2018-12-20

**Authors:** Sara Rocha, Joana Carvalho, Patrícia Oliveira, Maren Voglstaetter, Domitille Schvartz, Andreas R. Thomsen, Nadia Walter, Richa Khanduri, Jean‐Charles Sanchez, Andreas Keller, Carla Oliveira, Irina Nazarenko

**Affiliations:** ^1^ i3S—Instituto de Investigação e Inovação em Saúde Universidade do Porto Rua Alfredo Allen 208 4200‐135 Porto Portugal; ^2^ Ipatimup—Institute of Molecular Pathology and Immunology University of Porto Rua Júlio Amaral de Carvalho 45 4200‐135 Porto Portugal; ^3^ ICBAS—Instituto de Ciências Biomédicas Abel Salazar Universidade do Porto R. Jorge de Viterbo Ferreira 228 4050‐313 Porto Portugal; ^4^ Institute for Infection Prevention and Hospital Epidemiology Medical Center—University of Freiburg Faculty of Medicine University of Freiburg Breisacherstr. 115b 79106 Freiburg Germany; ^5^ Department of Human Protein Sciences Centre Médical Universitaire Rue Michel‐Servet 1 CH 1211 Geneva Switzerland; ^6^ Department of Radiation Oncology Medical Center—University of Freiburg Hugstaetterstr 55 Freiburg 79106 Germany; ^7^ German Cancer Consortium (DKTK) Partner Site Freiburg and German Cancer Research Center (DKFZ) Im Neuenheimer Feld 280 69120 Heidelberg Germany; ^8^ Clinical Bioinformatics University Hospital Saarland University Kirrberger Straße, Building E2.1 66123 Saarbrücken Germany; ^9^ Department Pathology and Oncology Faculty of Medicine University of Porto Alameda Prof. Hernâni Monteiro 4200‐319 Porto Portugal

**Keywords:** 3D cell culture, cancer, extracellular vesicles, integrative network analysis, microwell arrays

## Abstract

The success of malignant tumors is conditioned by the intercellular communication between tumor cells and their microenvironment, with extracellular vesicles (EVs) acting as main mediators. While the value of 3D conditions to study tumor cells is well established, the impact of cellular architecture on EV content and function is not investigated yet. Here, a recently developed 3D cell culture microwell array is adapted for EV production and a comprehensive comparative analysis of biochemical features, RNA and proteomic profiles of EVs secreted by 2D vs 3D cultures of gastric cancer cells, is performed. 3D cultures are significantly more efficient in producing EVs than 2D cultures. Global upregulation of microRNAs and downregulation of proteins in 3D are observed, indicating their dynamic coregulation in response to cellular architecture, with the ADP‐ribosylation factor 6 signaling pathway significantly downregulated in 3D EVs. The data strengthen the biological relevance of cellular architecture for production and cargo of EVs.

## 1 Introduction

Epithelial tumors account for 90% of the tumors in all organs^1^ and the success of tumor initiation and progression ultimately depends on the dynamic interactions taking place between the tumor cells and their microenvironment.^2^, ^3^ Recent findings have shown that extracellular vesicles (EVs) act as key mediators of this crosstalk.^4^, ^5^, ^6^ Depending on their subcellular origin, various types of EVs, different in their content and function, can be distinguished, such as exosomes (vesicles of endosomal origin in the size range from 50 to 150 nm), microvesicles (vesicles budding from the cell surface in the size range from 100 to 500 nm), and oncosomes (large vesicles of 1–10 µm in diameter produced by some tumors).^7^, ^8^ EVs serve as a universal mechanism of intercellular communication, transporting genetic information and bioactive molecules such as DNA,^9^, ^10^, ^11^ coding and noncoding RNA,^12^, ^13^, ^14^ proteins,^15^, ^16^ and lipids^17^, ^18^ from donor to recipient cells.^19^ The information conveyed by tumor‐derived EVs may functionally influence recipient cells and foster their reprogramming,^20^ thus contributing to the regulation of different stages of tumor progression.^21^, ^22^, ^23^, ^24^, ^25^ Therefore, once released into the circulation, EVs and EV‐associated molecules can be used as biomarkers in diagnosis,^26^, ^27^, ^28^ prognosis,^29^, ^30^ and therapeutic follow‐up of cancer patients.^31^, ^32^ The role of EVs in tumor progression has been addressed using mostly in vivo and conventional 2D in vitro models.^22^, ^24^ In these studies, EVs have been isolated either from biological fluids of cancer patients or, more often, from 2D cell cultures of cancer cells.^33^, ^34^ However, it is widely accepted that under 2D conditions, cells lack the in vivo spatial polarization and architecture, intrinsically related to their function, as well as cell–cell and cell–matrix interactions.^35^, ^36^ Moreover, 2D sources of EVs are poorly efficient as a large input volume is needed and the yield of isolated EVs is normally low. Altogether, these facts may hamper the scale‐up of 2D sources of EVs and their clinical implementation. In the last few years, 3D in vitro models have been developed and tailored to better recapitulate the in vivo features of tumors.^37^, ^38^, ^39^, ^40^, ^41^ In fact, it has been demonstrated that tumor cells growing in 3D conditions acquire morphology,^42^, ^43^ differentiation,^44^ growth pattern,^40^, ^45^ and gene expression signatures similar to those observed in human tumors.^46^ Importantly, it has already been shown that some of these cellular features, such as differentiation^47^ and phenotype,^48^, ^49^ affect the content and function of EVs. However, the impact of 3D cellular architecture on the production, content, and function of EVs remains to be elucidated.^50^, ^51^, ^52^ Additionally, it is unclear whether 3D models provide an efficient and cost‐effective production of EVs as compared to 2D models.

In this work, we addressed these questions by studying the differences and commonalities between EVs released by tumor cells growing in 2D and 3D cultures. For this purpose, we used a customized 3D in vitro model^53^ to isolate EVs released by cell aggregates from two gastric cancer (GC) cell lines. Thereafter, we performed a comprehensive comparative analysis of the biochemical features, small RNA transcriptome and proteome of EVs released by cells growing in 2D and 3D conditions. We found that GC cells cultured in 3D produce higher amounts of smaller EVs, as compared to the same cells grown in 2D. The small RNA profiles of EVs produced in 2D and 3D conditions were similar; however, there were specific microRNA signatures distinguishing EVs released in these two conditions. Moreover, EVs obtained from 2D and 3D cultures exhibited different protein expression profiles, showing significant downregulation of proteins assigned to the ADP‐ribosylation factor 6 (ARF6) signaling pathways in 3D. We also observed increased association between cells and EVs, and invasion capacity of recipient cells upon treatment with EVs derived from 3D cultures of a specific tumor cell line, without affecting proliferation and viability. Analysis of microRNAs and proteins differentially expressed under 2D and 3D conditions showed a global upregulation of microRNAs and downregulation of proteins in 3D. Integrative network analysis of these data revealed a dynamic coregulation of microRNAs and proteins in cells and EVs associated with the change of cellular architecture from 2D to 3D.

Altogether, these results indicate that the 3D cellular architecture modifies production levels and cargo of EVs, which may affect efficiency of association and consequently uptake and induce different functional behavior on recipient cells, rendering 3D cultures a more physiological high‐throughput alternative to conventional 2D in vitro systems.

## 2 Results and Discussion

### 2.1 Establishment and Characterization of 3D Microwell Array Culture of GC Cells

**Figure 1:**
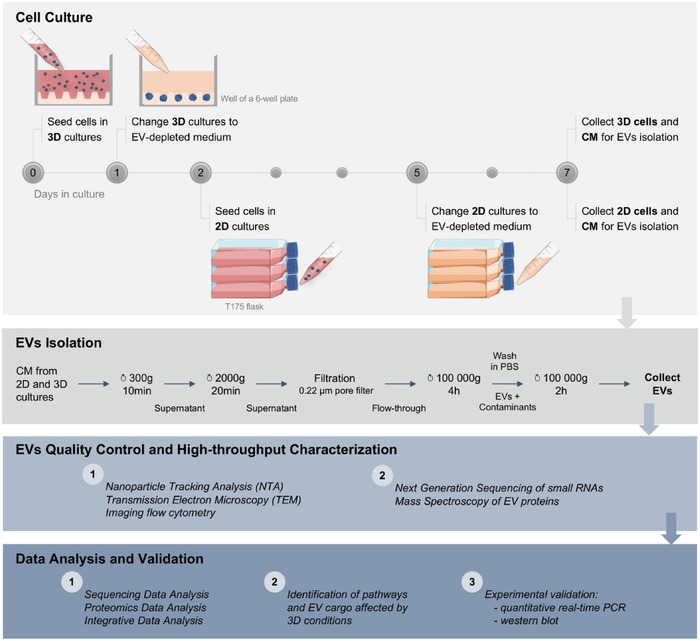
Scheme of the experimental flow for EV production, isolation, characterization, and data analysis. Conditioned medium (CM) isolated after 6 d of 3D cell culture and 2 d of 2D cell culture was used for EV isolation by differential centrifugation. Quality control techniques (NTA, TEM, and imaging flow cytometry) were performed on the isolated EVs prior to next‐generation sequencing of small RNAs and mass spectrometry of proteins. Resulting data were analyzed separately and further integrated for the identification of altered pathways and EV cargo in 3D cell culture conditions. Results were validated using independent samples and technical approaches (quantitative real‐time PCR and Western blotting).

To dissect a potential influence of 3D cellular architecture on the release and cargo of EVs, we designed and carried out a comprehensive study according to the scheme presented in **Figure** **1**.

**Figure 2:**
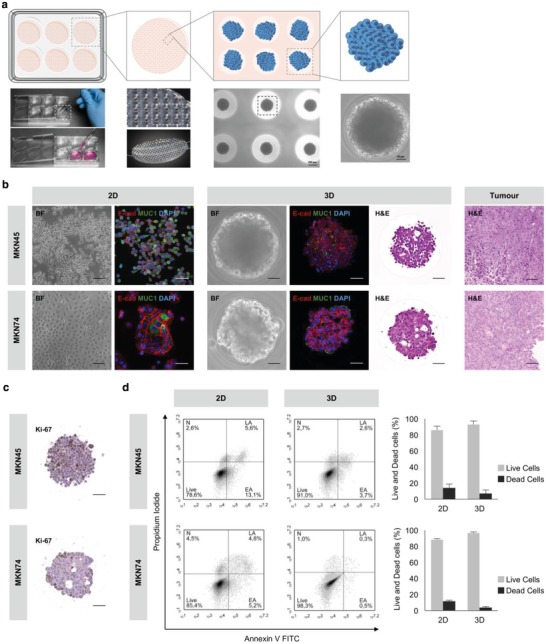
3D culture of GC cells resembles the native GC histological subtype. a) Illustration of the 3D culture method. Agarose microwell arrays with 1000 conical microwells were placed in six‐well plates and 1 × 10^6^ GC cells were seeded on the top of each matrix. GC aggregates, formed by liquid overlay, were allowed to grow for 6 d under EV‐depleted medium. b) Representative images of the morphology (BF), expression of epithelial markers (E‐cad/MUC1) of 2D and 3D cultures, and histology (H&E) of 3D cultures and xenograft tumors of GC cells. Scale bars: 50 µm. BF: bright field; Ecad/MUC1: E‐cadherin/Mucin‐1; H&E: hematoxylin and eosin staining. Xenograft tumors were obtained from subcutaneous injection of 1 × 10^6^ MKN45 cells and 5 × 10^6^ MKN74 in athymic nude mice. c) Ki‐67 staining showing representative proliferative patterns in 3D cultures. Scale bars: 50 µm. d) Viability of cells harvested from 2D and 3D cultures (dissociated spheroids) tested by flow cytometry of annexin V and propidium iodide. Data representative of five independent experiments are shown. N: necrotic cells; LA: late apoptotic cells; EA: early apoptotic cells; Live: live cells.

First, we established a new method for EV production using a recently developed and intensively characterized agarose microwell array,^53^ which allowed long‐term culture of cell spheroids and aggregates (**Figure** **2**a).

In this system, MKN45 and MKN74 GC cells grew in aggregates with a maximum size of 200 µm when seeded at a density of 1 × 10^5^ cells mL^−1^ and cultured for 6 d in EV‐depleted medium, without replenishment (Figure S1a,b, Supporting Information). While MKN45, a poorly differentiated cell line derived from GC liver metastases,^54^ formed aggregates of cells loosely attached to each other (Figure 2b, upper panel), MKN74, a moderately differentiated tubular adenocarcinoma cell line,^54^ formed compact cell aggregates with a glandular‐like organization (Figure 2b, bottom panel). Next, we examined the expression of E‐cadherin and Mucin‐1, proteins natively located at the basolateral and apical surfaces of normal gastric epithelial cells, respectively.^55^ MKN45 and MKN74 cells displayed expression of E‐cadherin at the basolateral membrane in both 2D and 3D cultures, resembling its physiological localization (Figure 2b). Mucin‐1 is frequently upregulated in invasive GC^56^ and its expression has been correlated with worse prognosis and tumor differentiation.^57^ In agreement with these observations, Mucin‐1 was only expressed at the apical localization in MKN74 cells cultured in 3D conditions (Figure 2b), suggesting that in 3D this differentiated cell line displays a phenotype that is similar to the one observed in tumors. Since MKN45 cell line was derived from a poorly differentiated tumor metastasis, apical Mucin‐1 expression was expected neither in 2D nor in 3D conditions. Furthermore, hematoxylin–eosin (H&E) staining showed phenotypical similarities between the structure of cell aggregates grown in microwell arrays and the structure of in vivo tumors, grown as xenografts in mice (Figure 2b, right panel). All these findings corroborated previous results using an in vitro multicellular gastric cancer spheroid model based on liquid overlay technique.^58^ To estimate proliferation, we performed Ki‐67 staining and detected proliferating cells in both MKN45 and MKN74 cell aggregates (Figure 2c). Cellular viability, measured after spheroid dissociation into single cell suspension, was determined by propidium iodide (PI)/annexin V staining. We observed that the percentage of viable cells was higher than 90% (Figure 2d), thus reducing the potential contamination of EV preparations by apoptotic bodies.^59^ Of notice, the percentage of dead cells growing in 2D conditions was already above 10% (Figure 2d). This result combined with the fact that cells grow fast and become overconfluent latest after day 5 prevents longer periods of 2D cultures.^59^

The high cell viability of 3D cultures suggested the absence of a hypoxic or necrotic core, which was expected since the maximal aggregate diameter observed was ≈200 µm.^60^ It is noteworthy that larger spheroids could be obtained, by increasing cell density at seeding, to mimic the hypoxic/necrotic core that characterizes tumors.

Altogether, these results indicate that the 3D microwell array is a suitable system for the culture of GC cells. Knowing that differentiation and polarization play a role in the regulation of EV release and content,^61^, ^62^, ^63^ and that GC aggregates reproduced better the properties of in vivo tumors, one may speculate that EVs produced in this 3D system may be closer to those produced by patient tumors, compared with standard 2D culture systems. Therefore, this system may constitute a useful model for EV production and functional studies under a more physiological environment.

### 2.2 3D Cellular Architecture Favors the Release of Smaller EVs Expressing Exosomal Markers

**Figure 3:**
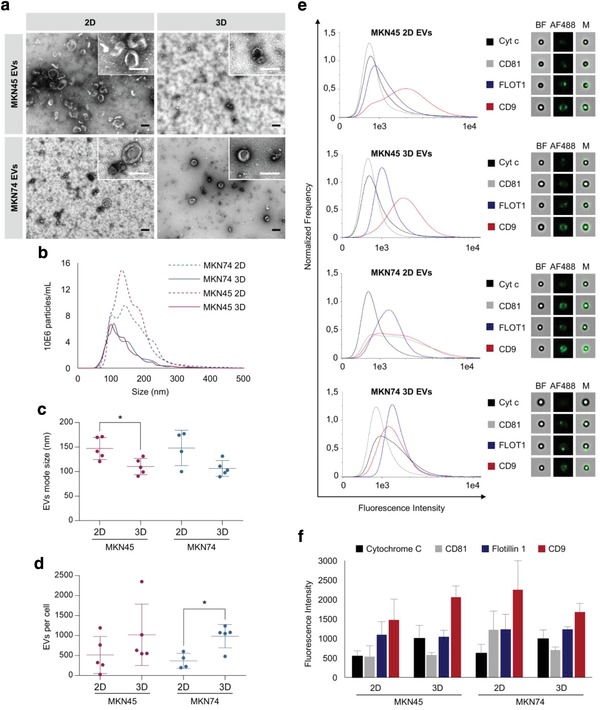
Increased EV production by GC cells growing in 3D cultures. a) Representative electron microscopy images of EVs isolated from 2D and 3D GC cultures. Scale bars: 100 nm. b–d) NTA of isolated EVs. b) Distribution of size, c) mode size, and d) number of EVs per cell; graphs represent the mean ± standard deviation of at least four biological replicates. The number of EVs per cell represents the ratio between the total number of isolated EVs and the total number of cells retrieved from the respective cultures. *p < 0.05, Mann–Whitney test. e,f) Detection of CD9, CD81, and Flotillin‐1 by imaging flow cytometry. e) Distribution and representative images of the intensity of fluorescence detected for each marker. Bright‐field images (BF) showed beads to which EVs were coupled; fluorescence images (AF488) showed EVs labeled with specific markers; merged images (M) showed labeled EVs coupled to beads. f) Quantitative analysis of the intensity of fluorescence of each marker. Results represent the mean ± standard deviation of at least three biological replicates.

To understand the impact of the 3D cellular architecture on the physical and biochemical properties of EVs, we performed transmission electron microscopy (TEM), nanoparticle tracking analysis (NTA), and imaging flow cytometry on EVs isolated by ultracentrifugation from 2D and 3D cultures. TEM revealed vesicular structures of a size typically assigned to exosomes and microvesicles (**Figure** **3**a).^8^ Additionally, EVs derived from 3D cultures tended to be smaller than EVs isolated from 2D cultures, which was further confirmed by NTA (Figure 3b,c). While EVs derived from 3D cultures had mean sizes between 85 and 135 nm, EVs isolated from 2D cultures had mean sizes of 100–180 nm (Figure 3b,c). By normalizing the total number of EVs isolated in each experiment to the total number of cells producing these EVs (cells detached from 2D monolayers or dissociated from 3D aggregates), we observed that the average number of EVs per cell was significantly higher in 3D in comparison to 2D (Figure 3d; Table S1, Supporting Information). This result may reflect two key parameters of the culture method: culture time and cell number. Whereas in 2D the maximum period for conditioned media collection was 48 h, in 3D this period could be extended for another 4 d without compromising cell viability. Moreover, in 3D cultures the number of cells used for producing EVs is considerably lower than that in 2D (Table S1, Supporting Information): threefold at the seeding and six‐ to ninefold at the end point, depending on the cell line. The volume of media required for 3D cultures was only 30% compared with 2D conditions. Altogether, these observations strengthened the efficiency of the 3D system.

These findings were in agreement with previous studies showing that exosomes released by tissue‐engineered tumors were smaller than exosomes released by the same cells cultured as monolayers, and had similar size distribution and concentration as exosomes isolated from human patient plasma.^52^ As EV preparations were enriched in small‐sized vesicles corresponding to the size of exosomes,^64^ we tested the expression of exosome markers CD9, Flotillin‐1, and CD81 (Cytochrome c—as a negative control) by imaging flow cytometry (Figure 3e,f; Figure S2, Supporting Information). We observed that EVs from 2D and 3D cultures harbored comparable amounts of CD81 and Flotillin‐1, while the portion of CD9‐positive EVs tends to be increased in MKN45 3D cultures (Figure 3f). Taken together, these results showed that GC cell lines cultured in 3D conditions are highly efficient in producing EVs that maintain the expression of exosomal markers.

### 2.3 EVs Isolated from 2D and 3D Cultures Exhibit Similar Small RNA Profiles

**Figure 4:**
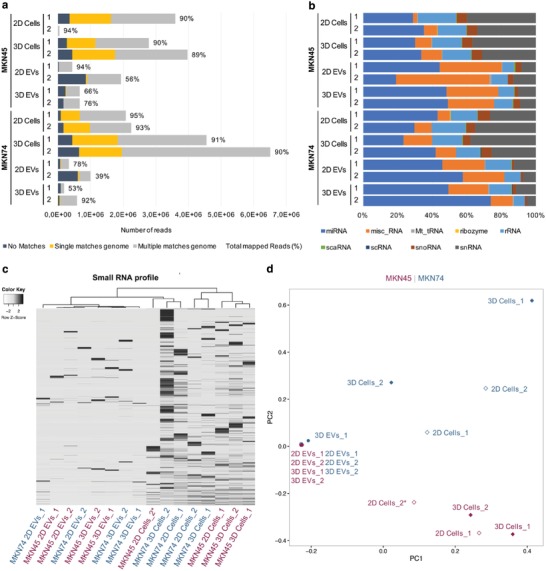
EVs released by GC cells under 2D and 3D conditions exhibit similar small RNA profiles. a) Total number of reads and percentage of mapped reads detected by small RNA sequencing. Reads that could not be mapped in the genome are shown in black; unique mappable reads are shown in dark gray; and reads that were mapped to multiple regions are shown in light gray. b) Distribution of mapped reads by small RNA classes. c) Heatmap and dendrogram of small RNA profiles of MKN45 and MKN74 EVs and cells in 2D and 3D cultures (*Z*‐score normalized expression values). d) Principal component analysis of small RNA profiles of MKN45 and MKN74 EVs and cells in 2D and 3D cultures. 1 and 2 represent two independent biological replicates. *Sample with reduced number of reads.

To evaluate the impact of 3D cellular architecture on the small RNA profile of EVs, we generated and sequenced small RNA libraries from EVs and their donor cells. Since characterization of the small RNA content of EVs is still accompanied by a number of technical challenges, we followed the recommendations of the International Society of Extracellular Vesicles for the processing of EVs for RNA isolation, quality control, and sequencing analysis.^65^ Of notice, the sequencing of one of the biological replicates of MKN45 2D cells was less efficient, exhibiting a lower number of reads. To overcome this, findings from one biological replicate were considered to be sufficient for all described comparisons. Comprehensive sequencing analysis of cellular and EV small RNAs revealed that more than 89% of the reads detected in cellular RNA and 39–94% of the reads in EV RNA mapped to the reference genome (**Figure** **4**a).

Notably, the vast majority of mapped reads from EV libraries corresponded to reads that mapped to multiple genomic locations, whereas cellular libraries also encompassed sequences mapped to single genomic regions (Figure 4a). To dissect the small RNA profile of each sample, we assigned the mapped reads to the different small RNA classes using the biotypes defined by Ensembl and miRBase. On average, we detected the expression of 303 small RNAs in EVs and 1675 in cellular samples (Table S2, Supporting Information). Of the small RNAs detected (fragments per kilobase of exon per million fragments mapped (FPKM) > 0), microRNAs were the most abundant, both in EVs and in cellular samples, comprising on average 54% and 41%, respectively (Figure 4b).

In fact, other studies have reported that the majority of small RNAs detected in EVs were assigned to microRNAs.^66^ Interestingly, our study used a next‐generation sequencing (NGS) platform distinct from the ones used in such studies, which goes against the notion that the NGS platform may introduce bias in the RNA biotypes identified.^67^ We also observed that while the second most abundant class of small RNAs in EVs was miscellaneous RNAs, in cells the second most abundant class was small nuclear RNAs (snRNA), comprising on average 26% and 29%, respectively (Figure 4b). Moreover, the high proportion of other types of small RNAs enclosed by EVs may reflect the different origins of EVs as described elsewhere.^67^, ^68^, ^69^ The sorting of all these small RNAs into EVs remains unclear; hence, future investigations should focus on understanding how different types of small RNAs are recruited to EVs. By hierarchical clustering, we observed that the expression profiles of small RNAs were not affected by the culture condition, neither among cells nor among EVs (Figure 4c). Principal component analysis revealed striking differences between small RNAs detected in EVs and in donor cells. While the small RNA profiles of all EVs samples exhibited high similarity, strong differences were observed between small RNA profiles of cells (Figure 4d).

### 2.4 EVs and Donor Cells from 2D and 3D Cultures Exhibit Similar Small RNA Profiles

**Figure 5:**
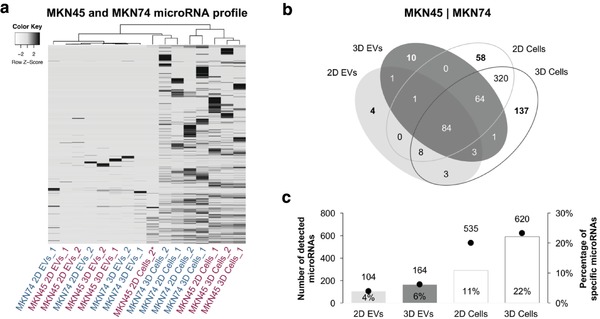
EVs and cells cultured under 2D and 3D conditions display distinct microRNA repertoires. a) Heatmap and dendrogram of microRNA profiles of GC EVs and cells in 2D and 3D cultures (*Z*‐score normalized expression values). b) Venn diagrams showing the distribution of detected microRNAs in both cell lines. c) Plots showing the number of total microRNAs and percentage of specific microRNAs detected in each condition (two biological replicates for each cell line).

Since microRNA was the most prevalent biotype identified in both EVs and cells, we further explored the microRNA expression profiles of our samples (**Figure** **5**; Figure S3a–f, Supporting Information). Using unsupervised hierarchical clustering, we observed no major differences between cells or EVs derived from 2D and 3D cultures, meaning that clusters were independent of culture conditions (Figure 5a). Next, we investigated whether a different cellular architecture affected specific microRNA signatures, by searching for microRNAs exclusively present either in cells or in EVs, derived from 2D and 3D cultures. We identified 58 microRNAs exclusively expressed in cells from 2D cultures and 137 microRNAs exclusively expressed in cells from 3D cultures (Figure 5b). Whereas most microRNAs detected in EVs were also present in cells, we identified four microRNAs specific for EVs isolated from 2D cultures and ten microRNAs specific for EVs isolated from 3D cultures. A single microRNA was shared by EVs from 2D and 3D cultures and not by cells (Figure 5b). Interestingly, these specific microRNAs, which discriminate spatial condition (2D vs 3D) and entity (cells vs EVs), corresponded to a small percentage of the total number of detected microRNAs (Figure 5c). The observation that EVs harbored a specific set of microRNAs, distinct from donor cells, was also seen in a breast cancer model, suggesting that it might be a general phenomenon in tumor‐derived EVs.^67^ Moreover, we observed that a set of 64 microRNAs was shared by cells from 2D and 3D cultures and present only in EVs derived from 3D cultures (Figure 5c). Of notice, the number of specific microRNAs present in cells or in EVs from 3D cultures was higher than the number of microRNAs detected exclusively in 2D (Figure 5c). Although experimental data explaining this phenomenon are scarce, sorting of certain microRNAs to EVs may require specific RNA‐binding proteins and recognition of specific sequence motifs.^70^

To explore the biochemical categories and pathways significantly enriched among the set of microRNAs present in EVs from 3D cultures, we performed an over‐representation analysis (ORA) of two groups of microRNAs using the miEAA tool.^71^ First, from the ten microRNAs exclusively present in EVs from 3D cultures (Figure 5b), hsa‐miR‐155‐5p, hsa‐miR‐143‐3p, and hsa‐miR‐127‐3p were significantly enriched in 48 categories, mostly associated with disease state, e.g., lymphoma, KRAS pathway, hypermethylation, and inflammatory response (Table S3, Supporting Information).

Next, from the group of 64 microRNAs shared by cells growing in 2D and 3D conditions and present only in EVs derived from 3D cultures (Figure 5b), six significant categories were obtained (Table S4, Supporting Information). The lowest *p*‐value of 0.01 was determined for 5/64 miRNAs, targeting the PIP5K1A signaling pathway, involved in the regulation of phosphoinositide metabolism, which plays a role in cell differentiation^72^ and invasion^73^ (Table S4, Supporting Information).

To identify pathways enriched among the 164 microRNAs detected in EVs from 3D cultures, regardless of their presence in EVs from other conditions, we performed a gene set enrichment analysis (GSEA), adapted for microRNAs (Table S5, Supporting Information).^71^ Among the top 20 hits, main cancer‐associated signaling pathways p53, MAPK, TGF‐β, and RAS were identified (Figure S4, Supporting Information).

Finally, to validate the NGS data, we quantified six randomly selected microRNAs, detected in both cells and EVs, using quantitative real‐time PCR (qRT‐PCR; Figure S5a–c, Supporting Information). We observed a strong correlation between the qRT‐PCR results and the small RNA sequencing data in cellular RNA, showing higher levels of the selected microRNAs in 3D samples (Figure S5d, Supporting Information). For EVs, the qRT‐PCR data correlated better in MKN74 than in MKN45 cell line, which in general exhibits a high level of plasticity and phenotypic heterogeneity (Figure S5d, Supporting Information).

### 2.5 ARF6 Signaling Pathway Is Downregulated in EVs Isolated from 3D Cultures

**Figure 6:**
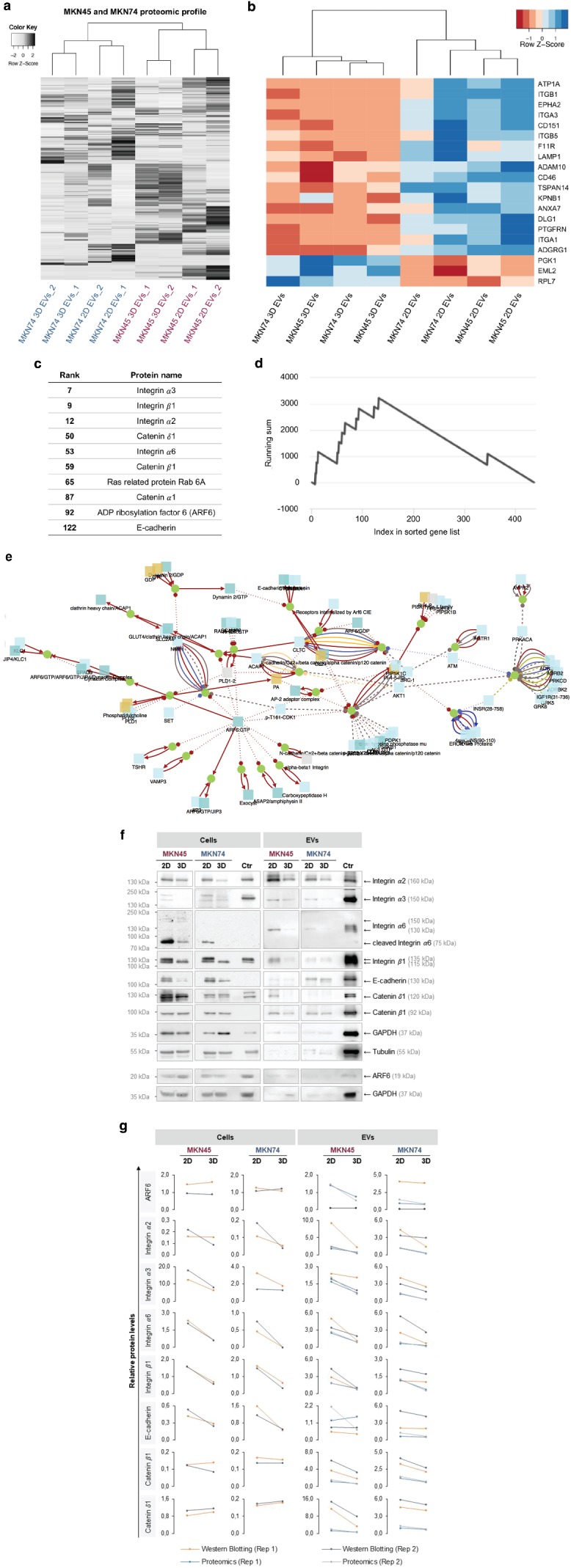
Protein set enrichment and network analysis show ARF6 signaling pathway significantly altered in EVs secreted by 3D cell cultures. a) Heatmap and dendrogram of protein profiles of GC EVs in 2D and 3D cultures (*Z*‐score normalized expression values). b) Supervised clustering was performed to highlight the differences between EVs secreted by 2D and 3D cultures. Heatmap and dendrogram of the 20 most significantly different proteins between EVs isolated from 2D and 3D cultures (*Z*‐score normalized expression values). c) Protein set enrichment analysis revealed ARF6 signaling pathway as the only pathway significantly altered between 2D and 3D conditions in both cell lines. The corresponding proteins were ranked according to the degree of deregulation between 2D and 3D. d) Running sum statistics highlighting the significant enrichment of the proteins listed. e) Representation of the ARF6 signaling network. f,g) Western blot validation and proteomics data of significantly altered proteins in 3D EVs. f) Representative Western blot images. g) Protein levels of significantly altered proteins quantified by densitometry analysis of Western blot and validation of proteomics data. Tubulin was used as endogenous control for cells and GAPDH for EVs. Each biological replicate was independently represented.

Next, we performed a comprehensive proteomics analysis of EVs released under 2D and 3D conditions by mass spectrometry (MS). In total, 430 proteins were identified in EVs released by MKN45 and MKN74 cells in 2D and 3D. Following normalization, unsupervised hierarchical clustering was tested to estimate the impact of EV origin (MKN45 vs MKN74) and spatial architecture (2D vs 3D). The protein expression profiles showed two separate clusters corresponding to MKN45 and MKN74 EVs, indicating that the cellular origin modulated the protein cargo of EVs (**Figure** **6**a). Moreover, within these clusters, a clear separation was detected corresponding to 2D and 3D conditions (Figure 6a; Figure S6, Supporting Information).

To highlight the differences between EVs released under 2D and 3D conditions, supervised clustering was applied to the 20 proteins with highest significance (Figure 6b). Remarkably, 17 out of 20 proteins had decreased expression in 3D compared to 2D, demonstrating an overall downregulation of proteins in EVs from 3D cultures (Table S6, Supporting Information). To unveil the proteins most affected by the spatial conditions, we applied protein set enrichment analysis (analogous to gene set enrichment analysis), listing proteins with respect to the degree of deregulation. We observed that the most downregulated proteins in 3D cultures are at the top of the sorted list (Figure 6c).

Next, we addressed whether proteins belonging to a certain biological category or pathway accumulate at the top or bottom of the sorted list by computing a running sum (Figure 6d). Using an algorithm relying on dynamic programming,^74^ we defined ARF6 signaling pathways to be significantly downregulated in EVs derived from 3D cultures (Figure 6e). ARF6 is a ubiquitously expressed protein involved in the regulation of membrane trafficking under physiological conditions and, together with syntenin, serves as one of the main regulators of exosome biogenesis.^75^, ^76^ In addition, it has been shown that ARF6 activation leads to an increased shedding of microvesicles from tumor cells, carrying proteases that may degrade the surrounding extracellular matrix.^77^ In fact, ARF6 upregulation/activation has been associated with an increase of cell motility and invasiveness in tumors.^78^ Taking into account that the proteomic profile of EVs from cells grown in the 3D system suggests downregulation of ARF6‐associated signaling pathways and knowing that these cells released higher numbers of smaller EVs, we may hypothesize that the cellular spatial architecture influences the biogenesis of EVs in an ARF6‐associated manner.

We validated seven proteins significantly downregulated in EVs from 3D cultures, by Western blotting, and a good correlation with proteomics data was observed (Figure 6f,g). Additionally, by evaluating these proteins also in the donor cells, we observed that all proteins except catenin β1 and catenin δ1 were downregulated in 3D (Figure 6f,g). Specifically for ARF6 protein, we observed no significant alterations at the protein levels in the donor cells (Figure 6g,h). Analysis of ARF6 in EVs by Western blotting confirmed results of proteomics data, showing downregulation of ARF6 in EVs from the MKN45 3D cultures (proteomics fold change: 0.41) and no differential expression of ARF6 in MKN74 EVs (proteomics fold change: 1.04; Figure 6g,h). These data suggested that the regulation of ARF6 expression may be cell line specific. However, taking into account the observed regulation of proteins involved in the ARF6 signaling pathway (Figure 6f,g), we cannot exclude other ARF6 regulatory mechanisms, such as its activation via phosphorylation,^79^ which were not addressed in our study. Therefore, the role of ARF6 and ARF6‐associated signaling pathways in the biogenesis and regulation of EV content, mediated by changes in cellular architecture, remains to be elucidated.

### 2.6 Dynamic Coregulation of MicroRNAs and Proteins in Response to Changes in Spatial Cellular Architecture

Given that in 3D cultures we observed an upregulation of microRNAs in cells and EVs (Figure 5), together with a downregulation of proteins in EVs (Figure 6), we hypothesized that changes in cell architecture induced a microRNA‐based regulation that targets cellular proteins, thus affecting EV cargo and their potential impact on recipient cells. Therefore, we analyzed the association between EVs derived from 2D and 3D cultures with different recipient cells: normal human dermal fibroblasts (NHDFs), immortalized normal epithelial cells (MCF10A), and the EV‐donor cancer cells MKN45 and MKN74 (Figure S7, Supporting Information). Furthermore, proliferation, viability, and invasion capacities of MCF10A treated with EVs isolated from MKN45 and MKN74 cancer cells growing in 2D and 3D conditions were also assessed (Figure S8, Supporting Information). We observed increased association during the first 15 min of the EVs derived from the 3D culture of the MKN45 cells when MCF10A and MKN45 were used as recipient cells. No difference between 3D and 2D conditions was obtained when testing MKN74 EVs (Figure S7, Supporting Information). The treatment with EVs from both cell lines did not change the proliferation and viability of recipient cells. Nevertheless, a significant increase in the invasion of recipient cells was induced by treatment with 3D EVs but not 2D EVs from the MKN45 cell line, indicating that despite similarity of cargo between EVs isolated from MKN45 and MKN74 3D cultures, their functional effect also relies on the inherited cell‐specific context.

**Figure 7:**
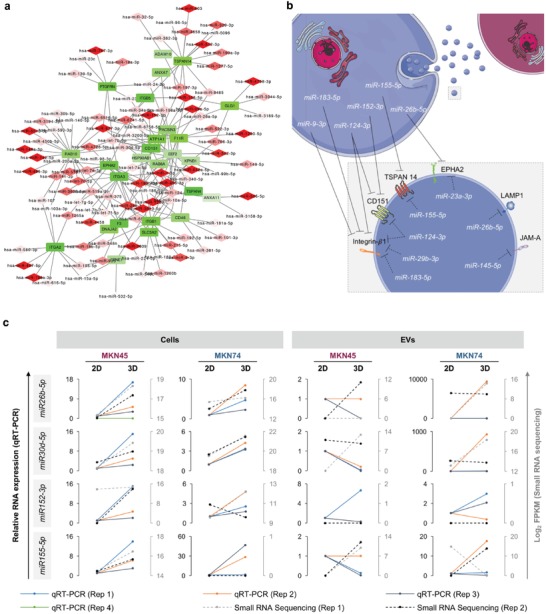
Integrative network analysis reveals overall upregulation of microRNAs and downregulation of proteins in EVs in 3D. a) Network of microRNAs (red rhombs) and specific target proteins (green rectangles) deregulated in opposite direction. Upregulation and downregulation in 3D conditions were represented in different scales of red and green, respectively. b) Schematic representation of dynamic coregulation of microRNAs in cells and EVs (white) and proteins in EVs (black) highlighting specific sets of miRNAs and their common target proteins in 3D conditions. c) Quantitative RT‐PCR validation and small RNA sequencing data of microRNAs of the integrative network in MKN45 and MKN74 cells and EVs. d,e) Western blot validation and proteomics data of proteins of the integrative network in MKN45 and MKN74 cells and EVs. d) Representative Western blot images for proteins of the integrative network in MKN45 and MKN74 cells and EVs. e) Relative levels of proteins of the integrative network quantified by densitometry analysis of Western blot data and proteomics. Tubulin was used as endogenous control for cells and GAPDH for EVs. c–e) Each biological replicate was independently represented.

Next, we performed an integrative network analysis of NGS and liquid chromatography–tandem mass spectrometry (LC–MS/MS) data to pinpoint clusters of microRNAs and proteins reflecting the 3D cellular architecture. For this, two main criteria were considered: 1) miRTarBase indicated strong or weak evidence that a microRNA targeted a specific protein and 2) the expression patterns of microRNAs and their target proteins were negatively correlated. With this analysis, several clusters were identified, three of which are depicted in Figure S9 in the Supporting Information. Of notice, such clusters may be constituted by more than one microRNA targeting the same protein.^80^ By combining the full set of significant clusters, we identified a network specific for 3D conditions (**Figure** **7**a,b).

Next, we selected four upregulated microRNAs (*hsa‐miR‐26b‐5p*, *hsa‐miR‐30d‐5p*, *hsa‐miR‐152‐3p*, and *hsa‐miR‐155‐5p*) and five downregulated proteins (ephrin receptor A2, CD151, junctional adhesion molecule A, importin β1, and lysosome‐associated membrane glycoprotein 1) for validation by qRT‐PCR (Figure 7c) and Western blot (Figure 7d,e). In cells, the expression levels of the four microRNAs correlated well with the data obtained by NGS (Figure 7c). In EVs, this correlation was maintained for MKN74 samples; however, the same was not observed for MKN45 samples (Figure 7c). Validation of protein data in EVs supported the postulated hypothesis, showing consistent downregulation of all selected proteins, with the exception of importin β1, which was not detected (Figure 7d,e).

## 3 Concluding Remarks

Our study focused on two main objectives: first, to establish a robust experimental system allowing efficient EV production under controlled conditions and closer to the physiological environment, in comparison with conventional 2D cultures; second, to understand the influence of cellular architecture on the release and content of EVs. Here, we described the application of a newly developed agarose microwell array for isolation of EVs released by long‐term culture of GC cell aggregates. Altogether, the high viability, the polarization, and morphology of cells grown in 3D and the increased number of obtained EVs empower the usage of this 3D system for scale‐up of EV production, allowing further high‐throughput experiments.

Using this system, we provided comprehensive evidence of the cellular architecture impact on EVs, including a comparative analysis of small RNAs and proteins present on EVs released by cells growing under 2D and 3D conditions, and functional assays.

Integrative network analysis of these data allowed us to raise the hypothesis of a dynamic coregulation of microRNAs and proteins, in cells and EVs, in response to changes on cellular spatial architecture. In summary, we observed that the 3D cellular architecture favored an upregulation of certain microRNAs, leading to the downregulation of the target proteins, thereby affecting the cargo of EVs released from cells.

Although the impact of spatial architecture in cell properties is widely accepted, this study is the first, to our knowledge, addressing the impact of 3D culture conditions on EVs. Our findings suggest that spatial architecture influences the biogenesis and cargo of EVs and that our 3D system represents an improvement for a more physiological, highly efficient, and cost‐effective production of EVs in comparison to conventional 2D in vitro systems. Understanding the relationship between cellular spatial organization and its consequences on the content and function of EVs will improve our knowledge on EV‐mediated intercellular communication and its implications in health and disease.

## 4 Experimental Section

*Cell Culture and Characterization*: Human GC cell lines MKN74 and MKN45 (ATCC, Manassas, VA, USA) were cultured in RPMI 1640 medium (Thermo Fisher Scientific, Waltham, MA, USA) supplemented with 10% fetal bovine serum (FBS; Biowest, Nuaillé, France) and 1% penicillin–streptomycin (PS; Thermo Fisher Scientific, Waltham, MA, USA). MCF10A cells (ATCC, Manassas, VA) were cultured in Dulbecco's modified Eagle medium:nutrient mixture F‐12 (DMEM/F12) supplemented with 5 µg mL^−1^ insulin, 1 µg mL^−1^ hydrocortisone, 5% horse serum, and 1 ng mL^−1^ epidermal growth factor. NHDFs were cultured in DMEM (low glucose), supplemented with 10% FBS (Biowest, Nuaillé, France). All cells were maintained at 37 °C in 5% CO_2_ humidified atmosphere.

EV‐depleted medium was obtained by overnight ultracentrifugation at 100 000 × *g* in a 70Ti rotor (Beckman Coulter, Fullerton, CA, USA) of RPMI medium supplemented with 20% FBS and 1% PS. This EV‐depleted medium was filtered through a 0.22 µm filter and further diluted in the same amount of RPMI medium supplemented with 1% PS and without FBS, to reach a final 10% FBS concentration.

For 2D cultures, GC cells were seeded in T175 flasks, and cultured in RPMI 1640 medium supplemented with 10% FBS and 1% PS until a confluence of 60–70% was reached. Next, GC cells were washed with phosphate buffer solution (PBS; Thermo Fisher Scientific, Waltham, MA, USA) and cultured in EV‐depleted medium (10% FBS final concentration) during 48 h. The conditioned medium was used for EV isolation and cells for downstream analysis. Cells growing in monolayer were detached using TrypLE Express Enzyme (Thermo Fisher Scientific, Waltham, MA, USA; 5 min, 37 °C), resuspended in normal medium and counted.

For 3D cultures, GC cells were seeded in customized microwell arrays developed at the Medical Center University of Freiburg,^53^ which are in the meantime commercially available as 3D CoSeedis (abc biopply, Switzerland). Cell seeding was performed at a density of 1 × 10^5^ cells mL^−1^, corresponding to ≈1 × 10^6^ cells per array and 1000 cells per microwell. GC 3D cultures were grown in RPMI medium supplemented with 10% FBS and 1% PS during the first day, and then cultured for 6 d in EV‐depleted medium. Optical microscopy was used to follow cell growth and spheroid formation. The conditioned medium was used for EV isolation and cells for downstream analysis. After spheroid dissociation using 0.5% Trypsin‐EDTA (Thermo Fisher Scientific, Waltham, MA, USA; 5 min, 37 °C), cells were resuspended in normal medium and counted.

*Histological Analysis and Immunofluorescence*: GC cells growing in 3D cultures were fixed overnight, at room temperature (RT), using 2% paraformaldehyde (PFA; Merck, Darmstadt, Germany) followed by Gill's hematoxylin (Bio‐Optica, Milan, Italy) staining for 10 min. Then, 2.4% low melting point agarose (50 °C; Lonza, Basel, Switzerland) was added on top of 3D cultures followed by gelling (10 min at RT and 20 min on ice). Paraffin sections of 3 µm were used for H&E, Ki‐67, and immunofluorescence staining.

For H&E staining, slides were incubated with hematoxylin for 4 min, rinsed in ethanol, and stained with eosin for 2 min.

For Ki‐67 staining, antigen retrieval was performed using citrate buffer (1:100 dilution, pH 6.0, at 98 °C, 40 min; Abcam, Cambridge, UK). Endogenous peroxidase activity was blocked using 3% hydrogen peroxidase solution (1:10 dilution, 20 min; Sigma‐Aldrich, St. Louis, MO, USA) followed by incubation with anti‐Ki‐67 antibody (SP6, 1:200 dilution, 90 min; Thermo Fisher Scientific, Waltham, MA, USA). After washing, slides were incubated with REAL EnVision Detection System (Dako, Glostrup, Denmark) substrate buffer (30 min) and with DAB Chromogen (10 min).

For immunofluorescence, samples from 2D and 3D cultures were costained for E‐cadherin and Mucin‐1. 2D cultures were fixed in 4% PFA (20 min), followed by treatment with NH_4_Cl 50 × 10^−3^ m (10 min; Merck, Darmstadt, Germany) and Triton X‐100 0.2% (5 min; Sigma‐Aldrich, St. Louis, MO, USA) and blocked with 5% BSA (30 min; NZYTech, Lisbon, Portugal). For 3D cultures, antigen retrieval was performed as described for Ki‐67 staining, followed by incubation in blocking serum (20 min; Ultra V Block; Lab Vision Corporation, Fremont, CA, USA). 2D and 3D samples were coincubated (at 4 °C, overnight) with rabbit anti‐E‐cadherin and mouse anti‐Mucin‐1 primary antibodies, followed by 1 h incubation with secondary antibodies anti‐rabbit Alexa 594 and anti‐mouse Alexa 488. Images were taken with a TCS‐SP5 AOBS confocal microscope (Leica Microsystems, Wetzlar, Germany) and slides were scanned using Axio Scan.Z1 slide scanner system (Zeiss, Oberkochen, Germany). See antibody details in Table S7 in the Supporting Information.

*Annexin V‐FITC/Propidium Iodide Staining*: Cell viability was measured using annexin V‐FITC and PI double staining followed by flow cytometry. Cells from 2D cultures were harvested using TrypLE Express Enzyme. Aggregates from 3D cultures were retrieved from the agarose matrix by centrifugation (3 min, at 300 × *g*) and dissociated using 0.5% Trypsin‐EDTA. Cells recovered from the centrifuged conditioned media (300 × *g* pellet—EV isolation protocol described below) were pooled with cells dissociated from 2D or 3D cultures prior to annexin V‐FITC/PI staining. Dissociated 2D and 3D cells were washed with annexin V binding buffer (AVBB; 10 × 10^−3^ m HEPES, 140 × 10^−3^ m NaCl, 2.5 × 10^−3^ m CaCl_2_) and stained with annexin V‐FITC (1:20, 15 min; ImmunoTools, Friesoythe, Germany). Cells were stained with PI (2 µg mL^−1^, 15 min; Thermo Fisher Scientific, Waltham, MA, USA), washed twice with AVBB, filtered through a 0.22 µm filter, and analyzed in a BD Accuri C6 flow cytometer (Becton, Dickinson and Company, Franklin Lakes, NJ, USA). Dead cells represent the sum of early apoptotic (annexin V‐FITC^+^/PI^−^), late apoptotic (annexin V‐FITC^+^/PI^+^), and necrotic (annexin V‐FITC^−^/PI^+^) cell populations. Live cells were considered to be annexin V‐FITC^−^/PI^−^. Results represent the mean ± standard deviation of three biological replicates of each cell line and culture condition.

*EV Isolation by Differential Ultracentrifugation*: EVs were isolated from the conditioned media of 2D and 3D cultures, growing in EV‐depleted medium, by differential centrifugation adapting previously described protocols.^59^ Briefly, the collected conditioned medium was centrifuged at 300 × *g*, 4 °C, for 10 min to pellet cells. The remaining supernatant was transferred to new tubes, centrifuged at 2 000 × *g*, 4 °C, for 20 min and filtered through a 0.22 µm filter. The filtered supernatant was further centrifuged in a SW32 rotor (Beckman Coulter, Fullerton, CA, USA) at 100 000 × *g*, 4 °C, for 4 h to pellet EVs. The EV pellet was washed in PBS without Ca^2+^ and Mg^2+^ (Capricorn Scientific GmbH, Ebsdorfergrund, Germany) and centrifuged at 100 000 × *g*, 4 °C, for 2 h before being resuspended in an appropriate volume of 0.9% NaCl, in accordance with downstream applications. Characterization of EVs is annotated in the EV‐TRACK Database as EV‐TRACK Nr. EV180053.

*Negative Staining and Visualization of EVs by Transmission Electron Microscopy*: The morphology of EVs was analyzed by TEM at the Histology and Electron Microscopy facility of i3S. In brief, an aliquot (10 µL) of fresh isolated EV preparations was mixed with an equal volume of 4% PFA, to make a final concentration of 2% PFA. Fixed EVs (5 µL) were then added to Formvar‐carbon‐coated grids and incubated for 1 min, in the dark at RT, to allow adsorption to the grid. Thereafter, EVs were stained with 5% uranyl acetate during 1 min and examined with a JEM 1400 electron microscope (JEOL, Tokyo, Japan). Images were recorded using a SC1000 Orius CCD camera (Gatan, Pleasanton, CA, USA).

*Nanoparticle Tracking Analysis of EVs*: The size and concentration of isolated EVs were measured by NTA, using the NanoSight NS300 instrument (Malvern, Worcestershire, UK) with scientific CMOS sensor. Samples were diluted in 0.9% NaCl to achieve a particle count between 1 × 10^8^ and 1 × 10^9^ particles mL^−1^. For each biological replicate, three technical measurements (videos) were recorded under controlled fluid flow with a pump speed set to 40. Camera focus was adjusted to give a clear sharp image of the particles (camera level between 10 and 16). The three videos of 30 s, with more than 1000 detected tracks per video, were analyzed using the automatic functions of NTA 3.1 Build 3.1.54 software and a detection threshold fixed at 5. Mode and mean size were calculated from the three videos per biological replicate. Results represent the mean ± standard deviation of at least four biological replicates of each cell line and were analyzed using the Mann–Whitney test (GraphPad Prism). Statistical significance was considered when *p* < 0.05.

*EV Characterization by Imaging Flow Cytometry*: EVs isolated from 2D and 3D cultures were analyzed for the expression of CD9, CD81, Flotillin‐1, and Cytochrome c using imaging flow cytometry. First, aldehyde/sulfate latex beads (4 µm, 4% w/v; Thermo Fisher Scientific, Waltham, MA, USA) were washed in PBS, spun down at 14 000 rpm for 3 min, 4 °C, and sonicated for 5 min on ice. EVs were then coupled to sonicated beads (1 × 10^9^ particles determined by NTA per 3 µL of beads) for 1 h at RT with agitation, resuspended in 1 mL PBS, and incubated overnight, at 4 °C. EVs coupled to beads (EVs–beads) were blocked in 100 × 10^−3^ m glycine solution (30 min; Merck, Darmstadt, Germany) prior to immunostaining. EVs–beads were labeled during 1 h with mouse monoclonal anti‐CD9, anti‐CD81, anti‐Cytochrome c, and rabbit polyclonal anti‐Flotillin‐1. After washing (PBS–2% BSA), labeled EVs–beads were incubated with goat anti‐rabbit/anti‐mouse Alexa Fluor 488 secondary antibodies (30 min) and analyzed by imaging flow cytometry. EVs–beads incubated only with secondary antibodies were used as negative controls.

Acquisition was performed using ImageStreamX Imaging Flow Cytometer (Amnis Corporation, Seattle, WA, USA) equipped with INSPIRE software. This technology was demonstrated to be able to prevent the acquisition of confounding signal derived from dye aggregates, which may have a size comparable to the used latex beads, allowing to improve accuracy of EV measurements.^81^ For each sample, 100 000 events were acquired at a 40× magnification. Fluorescence of the stained EVs–beads was excited with a 488 argon laser and collected on channel 2 (505–560 nm). A 745 nm laser was activated for side scatter and collected on channel 6, and bright‐field images with adjusted intensity were collected on channel 1. Data analysis was performed using the IDEAS software (Amnis Corporation, Seattle, WA, USA) using the pipeline described in Figure S2 in the Supporting Information. Data are shown as mean ± standard deviation. See antibody details in Table S7 in the Supporting Information.

*RNA Extraction*: Small RNA was isolated from 2D and 3D cells using mirVana miRNA isolation kit (Thermo Fisher Scientific, Waltham, MA, USA) according to manufacturer's instructions and including the final procedure for small RNA enrichment. For the isolation of small RNA from 2D and 3D EV preparations, mirCURY RNA isolation kit—Biofluids (Exiqon, Vedbaek, Denmark) was used, according to manufacturer's instructions. Prior to the small RNA isolation, EVs were treated with RNAse A (final concentration 0.4 mg mL^−1^, at 37 °C, 10 min; NZYTech, Lisbon, Portugal). RNAse A was inhibited with RNasin ribonuclease inhibitor (final concentration 1 U µL^−1^; Promega, Madison, WI, USA). Samples were treated with proteinase K (final concentration 0.05 mg mL^−1^, at 37 °C, 10 min; Qiagen, Hilden, Germany), and inactivated at 75 °C for 10 min. Concentration and quality of small RNAs, including microRNAs, were measured using the Agilent 2100 Bioanalyzer with the small RNA kit (Agilent, Santa Clara, CA, USA).

*Small RNA Library Preparation and Sequencing*: Two independent small RNA samples isolated from cells and EVs collected from 2D and 3D cultures of MKN45 and MKN74 cells were used for small RNA library preparation using the Ion Total RNA‐Seq Kit v2 (Thermo Fisher Scientific, Waltham, MA, USA), according to manufacturer's instructions. Briefly, 3′ and 5′ adaptors were attached directionally, and simultaneously, to 3 µL of small RNA input sample (≈10 ng of miRNA). Then, hybridized and ligated RNA was reversed transcribed using Ion RT primer v2 and SuperScript III Enzyme mix followed by purification and size selection with a magnetic bead–based method. Each cDNA sample was amplified and barcoded using Platinum PCR SuperMix High Fidelity, Ion Xpress RNA 3′ Barcode Primer, and a unique Ion Xpress RNA‐Seq Barcode BC Primer, which allowed sample identification and tracking. Amplified DNA was submitted to purification and size selection with a magnetic bead cleanup module and the yield and size distribution were assessed with Agilent 2200 TapeStation (S/N 3‐PM‐1173NA)—HS D1000 Screen Tape (P/N 5067‐5584). The small RNA barcoded libraries were diluted to the same molar concentration and an equal volume of each diluted library was used to prepare the final pool. Pooled libraries were processed on Ion Chef System (S/N CHEF00657) using the Ion 540 Kit‐Chef (P/N A27759) and the resulting 540 chip (P/N A27766) was sequenced on Ion S5 XL System (S/N 245717100156). FASTQ files were generated using the Torrent Suit plugin FileExporter v5.0.

*Small RNA Sequencing Data Analysis*: High‐quality reads from the resulting 16 FASTQ files were aligned to *Homo sapiens* reference genome (Assembly: GCA_000001405.15) using Bowtie2.^82^ Mapped reads were annotated using the corresponding GenBank information and miRBase (v.21).^83^ Expression data for each sample were calculated using Cufflinks^84^ and obtained in FPKM. *Cuffdiff* 84 was used to pinpoint differentially expressed genes for each described sample comparison. Private Perl scripts and Ensembl database^85^ information was used for expression data selection of genes with one of the following biotypes: miRNA, misc_RNA, mt_tRNA, ribozyme, rRNA, scaRNA, scRNA, snoRNA, and snRNA. Expression data from selected genes was used for: 1) heatmap construction using FPKM values scaled to *Z*‐score with the R package “gplots”^86^; 2) supervised and unsupervised hierarchical clustering for dendrogram construction using calculated Euclidean distances, using R package “gplots”^86^; and 3) Venn diagram plotting using jvenn.^87^ Of notice, microRNAs with FPKM > 0 were considered as expressed and used for Venn diagram plotting. Moreover, we considered a microRNA as expressed when detected in at least one of the biological replicates. RNA sequencing data will be available upon request.

ORA was performed using the miEAA tool.^71^ Ten microRNAs specifically present in 3D EVs and 64 microRNAs present in 3D EVs and in 2D and 3D cells were subjected to the analysis. As a reference list, we defined a total number of 164 microRNAs that were detected in all EVs from 3D culture. We considered all categories with nominal *p*‐values below 0.05 as significant but only considered categories containing at least two miRNAs. For a GSEA, the 164 miRNAs detected in EVs from 3D culture were ranked according to their average FPKM value and analyzed using the default settings in miEAA tool, such as significance value of 0.05 and threshold level of 2. The categories pathways, Gene Ontology, diseases, and targets were selected for the analysis.

*Quantitative Real‐Time PCR*: TaqMan Advanced microRNA assays (Thermo Fisher Scientific, Waltham, MA, USA) were used to quantify specific microRNAs on cellular and EV RNA samples, on a 7500 Fast Real‐Time PCR system (Thermo Fisher Scientific, Waltham, MA, USA). Analysis was performed using the cell and EV RNA samples, previously submitted to small RNA sequencing plus one independent biological replicate for MKN74 samples and two independent biological replicates for MKN45 samples. Briefly, 10 ng of RNA was reversed transcribed using universal RT primers and the TaqMan Advanced microRNA cDNA Synthesis Kit (Thermo Fisher Scientific, Waltham, MA, USA), according to manufacturer's instructions. Then, real‐time PCR was performed for each sample using 7 µL of diluted cDNA, TaqMan Advanced microRNA assays, and TaqMan Fast Advanced Master Mix (Thermo Fisher Scientific, Waltham, MA, USA) under fast cycling conditions. Reactions were done in triplicates and data were analyzed by the comparative 2^−ΔCT^ method, where ΔCT = *C*(*t*)_miRNA 3D cells_ − *C*(*t*)_miRNA 2D cells_ or ΔCT = *C*(*t*)_miRNA 3D EVs_ − *C*(*t*)_miRNA 2D EVs_. Results were represented as mean ± standard deviation.

Using the small RNA sequencing data, we searched for a small RNA that could be used as a housekeeping gene. Such small RNA must be expressed in all samples (FPKM > 0), with a good sample detection (FPKM > 10 000) and with no differential expression between samples (0.5 ≤ fold change ≤ 1.5). As we could not find a single candidate for normalization of qRT‐PCR data, we focused the analysis on the presence or absence of a certain microRNA, rather than its differential expression. Thus, individual replicate data were shown rather than the mean ± standard deviation. See microRNA assay details in Table S7 in the Supporting Information.

*Protein Sample Preparation for Tandem Mass Tag Labeling*: EV pellets were suspended in RapiGest 0.1% (in TEAB 0.1 m, pH 8). Volumes were adjusted to 100 µL with RapiGest 0.1% (in TEAB 0.1 m, pH 8). Reduction of 10 µg of protein per sample was done with tris(2‐carboxyethyl)phosphine at final concentration of 10 × 10^−3^ m; the samples reacted for 30 min at 60 °C. Alkylation was done with iodoacetamide, added to a final concentration of 40 × 10^−3^ m to each sample following incubation for 60 min, in the dark, at RT. Trypsin was added (ratio of 1:25, w/w), and the digestion was performed overnight at 37 °C. Then, a 10‐plex Tandem Mass Tags (TMT; Thermo Fisher Scientific) labeling was performed according to manufacturer's instructions. TMT reagent tags were dissolved in acetonitrile (ACN), and each sample was incubated for 60 min at RT with a specific tag. For TMT quenching, 8 µL of hydroxylamin 5% v/v was added, and incubated with the samples for 15 min. TMT‐labeled samples were pooled. For RapiGest cleavage, samples were incubated with TFA for 45 min at 37 °C (pH < 2). After centrifugation at 14 000 rpm for 10 min, supernatants were recovered and dried under vacuum. Samples were dissolved in 5% ACN/0.1% formic acid (FA) and desalted with C18 microspin columns (Thermo Fisher Scientific). Peptides were separated by off‐gel electrophoresis, desalted, and solubilized in an appropriate amount of 5% ACN/0.1% FA for MS analysis.

*Liquid Chromatography–Tandem Mass Spectrometry*: The peptides were dissolved in 5% ACN/0.1% FA to a concentration of 0.25 µg µL^−1^. Mass spectrometry experiments were performed on a Q Exactive Plus equipped with an Easy‐nanoLC (Thermo Fisher Scientific). Peptides were trapped on 2 cm × 75 µm ID, 3 µm precolumn and separated on an Easy‐spray column, 50 cm × 75 µm ID, PepMap C18, 2 µm (Thermo Fisher Scientific). The analytical separation was run for 60 min using a gradient of H_2_O/FA 99.9%/0.1% (solvent A) and CH_3_CN/FA 99.9%/0.1% (solvent B) at a flow rate of 300 nL min^−1^. For MS survey scans, the Orbitrap tandem (OT) resolution was set to 140 000 and the ion population was set to 3 × 10^6^ with an *m*/*z* window from 350 to 2000. Twenty precursor ions were selected for higher‐energy collisional dissociation with a resolution of 35 000, an ion population set to 1 × 10^5^ (isolation window of 0.5 *m*/*z*), and normalized collision energy set to 30%.

*Protein Identification and Quantification*: Raw data were loaded on Proteome Discoverer 2.1 software for identification and/or quantification of peptides and proteins. Identification was performed in the UniProt/SwissProt human database (2015_03, 20 203 entries) using Mascot (Version 2.5.1, Matrix Sciences, London, UK). Carbamidomethylation of cysteines, TMT‐sixplex amino terminus, and TMT‐sixplex lysine (for TMT‐labeled samples) was set as fixed modifications and methionine oxidation as variable modifications. Trypsin was selected as the enzyme, with two potential miss cleavages. Peptide and fragment ion tolerances were 10 ppm and 0.02 Da, respectively. Threshold of the average reporter signal‐to‐noise ratio was set to 1 and false discovery ratio was set to 1% at peptide–spectrum match, peptide, and protein levels. Only high‐confidence master proteins with at least two distinct peptide sequences were subjected to identification.

*Western Blotting*: Cells and EV pellets were lysed in HEPES lysis buffer (25 × 10^−3^ m HEPES, pH 7.4, 150 × 10^−3^ m NaCl, 5 × 10^−3^ m MgCl_2_, 1% Triton X‐100, 2 × 10^−3^ m phenylmethylsulfonyl fluoride, protease inhibitor tabs, EDTA‐free, Promega) for 45 min at 4 °C. After lysis, samples were centrifuged at 15 000 × *g*, 4 °C, for 20 min. For analysis, 30 µg of cell lysates and 20 µg of EV lysates were subjected to SDS‐PAGE followed by immunoblotting. SuperSignal West DURA Extended Duration Substrate (Pierce) was used for signal detection. See antibody details in Table S7 in the Supporting Information.

*Association of EVs and Recipient Cells*: The association between EVs and recipient cells was measured using flow cytometry. EVs released from the 2D and 3D MKN45 and MKN74 cultures were tested in this assay. As recipients MCF10A, NHDFs, and the EV donors, MKN45 and MKN74 cells, were tested. Recipient cells were seeded in 24‐well plates, and on the next day, when reaching 80% confluence, cells were incubated with EVs labeled with PKH26 (Sigma) according to the recommendations of the supplier. Briefly, 1.25 × 10^9^ EVs were brought to the final volume of 250 µL with the diluent C and mixed, 1 µL of PKH26 was mixed with 249 µL diluent C, and both solutions were mixed and incubated for 5 min in the dark. Staining was stopped with 500 µL 1% BSA. To remove unbound dye, the EVs were loaded in centrifugal filter units (100 kDa cutoff) and centrifuged for 5 min at 2500 ×*g* at RT. Flow‐through was discarded and EVs were brought to the final volume of 300 µL using the serum‐free medium. As a control, PBS without EVs was stained using the same protocol.

Prior to incubation of recipient cells with EVs, cells were washed with PBS, and 1.25 × 10^9^ labeled EVs were added to each well and incubated for 5, 15, or 30 min at 37 °C. After that EVs were removed and the cells were washed, trypsinized, and fluorescence intensity was measured by flow cytometry (BD Accuri C6) using thresholds FSC‐H 80.000, SSC‐H 10, flow rate 35 µL min^−1^, core size 16 µm, and 10 000 counts; for NHDFs, 6000 count limit was used.

*Functional Assays*: Cell invasion, proliferation, and viability of normal epithelial cells (MCF10A) were measured upon treatment with EVs derived from 2D and 3D cultures of MKN45 and MKN74 cells.

First, MCF10A cells were washed with PBS and harvested using Versene (Thermo Fisher Scientific) followed by staining with CellTrace Cell Proliferation Kit, accordingly to manufacturer's instructions (Thermo Fisher Scientific). Then, 5 × 10^5^ labeled cells were seeded and treated with 5 × 10^9^ EVs in the next day. After 24 h of treatment, cells were collected and submitted to different functional assays. For the invasion assay, Corning BioCoat Matrigel Invasion Chambers (Thermo Fisher Scientific) were hydrated with DMEM/F12 medium (Thermo Fisher Scientific) during 1 h at 37 °C. Then, 5 × 10^4^ cells were seeded in the upper compartment and incubated for 24 h at 37 °C in 5% CO_2_. Filters were washed in PBS and fixed in ice‐cold methanol for 10 min. Noninvasive cells and Matrigel were removed with a prewet cotton swab, and filters were washed and mounted in glass coverslips with Vectashield/DAPI. A mosaic of the entire filters was obtained with Axiovert 200M (Zeiss, Oberkochen, Germany) and the total number of invasive nuclei was counted using ImageJ software.

Cell proliferation was determined by analyzing CellTrace dilution using a BD FACSCanto II flow cytometer (Becton, Dickinson and Company, Franklin Lakes, NJ, USA). Cell viability was performed as mentioned earlier. Results represent the mean ± standard deviation of at least three biological replicates. Data were analyzed with a one‐way analysis of variance with Tukey's multiple comparisons test.

*MicroRNA Profiling and Proteomics Data Integration*: For proteomics data, protein set enrichment analysis was performed using the multiomics analysis toolkit GeneTrail2,^88^ freely available at https://genetrail2.bioinf.uni‐sb.de. Proteins were ordered with respect to the degree of deregulation and significance values were calculated by an exact algorithm relying on dynamic programming.^74^ Next, an integrative analysis of miRNAs and proteins was performed using the criteria described in the Results and Discussion section. Two criteria were considered: 1) miRTarBase indicated strong or weak evidence that a microRNA targeted a specific protein and 2) the expression patterns of microRNAs and their target proteins were negatively correlated. Of notice, miRTarBase strong evidence targets were those found by reporter assays, while weak evidence targets were derived from high‐throughput techniques such as microarrays.

## Conflict of Interest

The authors declare no conflict of interest.

## Supporting information

Supplementary
