# Supplementary material for: 3D Cellular Architecture Affects MicroRNA and Protein Cargo of Extracellular Vesicles
Source: Adv Sci (Weinh). 2018 Dec 20;6(4):1800948. doi: 10.1002/advs.201800948 (PMC6382357; doi:10.1002/advs.201800948)
Supplement: Supplementary file 1 — Supplementary [file ADVS-6-1800948-s001.pdf]

Copyright WILEY-VCH Verlag GmbH & Co. KGaA, 69469 Weinheim, Germany, 2018.

ADVANCED SCIENCE
Open Access

Supporting Information

for *Adv. Sci.,* DOI: 10.1002/advs.201800948

3D Cellular Architecture Affects MicroRNA and Protein Cargo of Extracellular Vesicles

Sara Rocha, Joana Carvalho, Patrícia Oliveira, Maren Voglstaetter, Domitille Schvartz, Andreas R. Thomsen, Nadia Walter, Richa Khanduri, Jean-Charles Sanchez, Andreas Keller, Carla Oliveira, and Irina Nazarenko**

Supplementary Figures

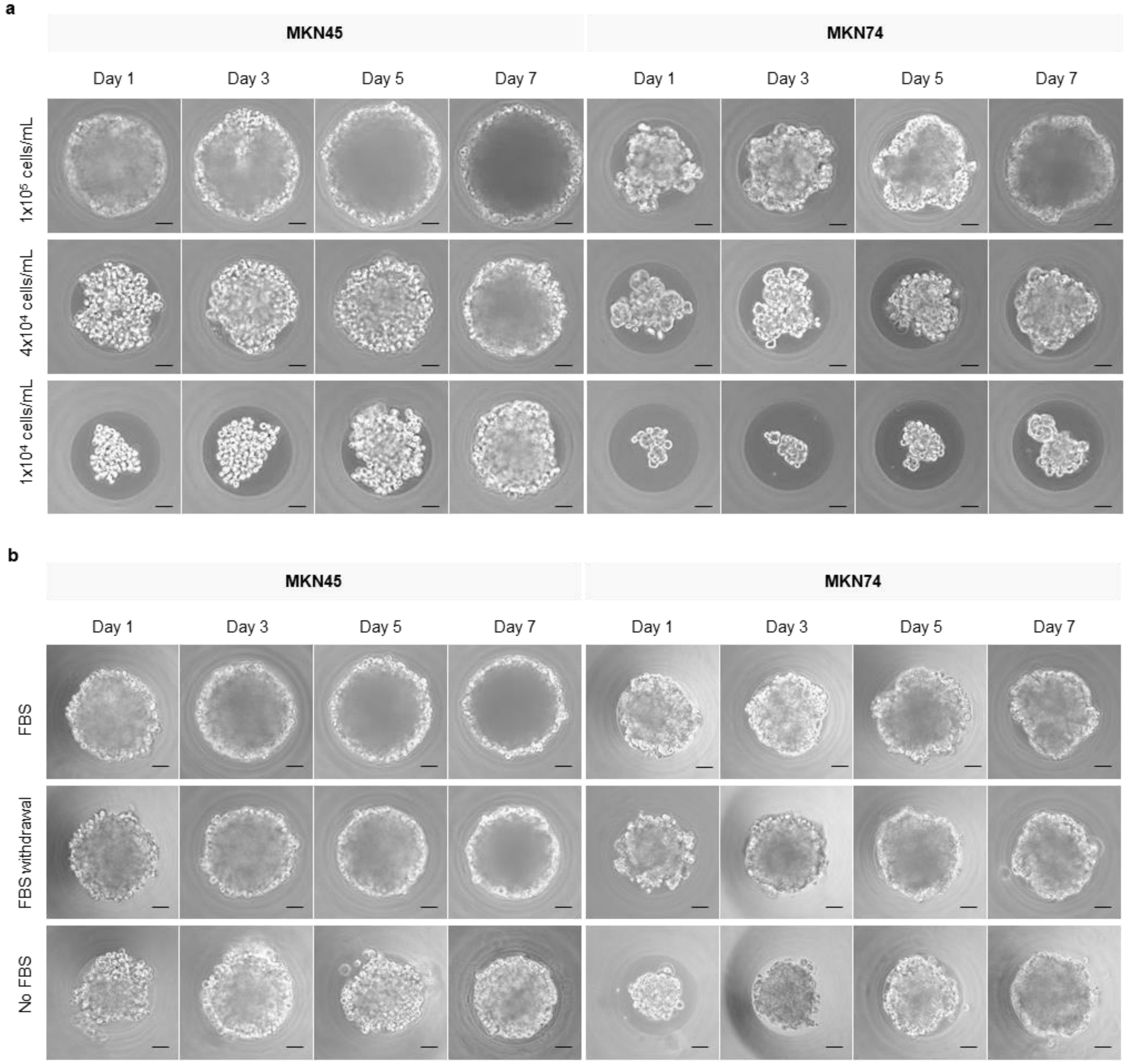

Supplementary Figure 1. Optimization of the 3D culture model. (a) Representative microscope images of aggregates obtained using different cell seeding densities**.** Cell seeding at a density of $1x10^5$ cells/mL ($1x10^6$ cells per agarose array) enables the establishment of larger aggregates (~200µm). **(b)** Representative microscope images of aggregates cultured with distinct FBS supplementations**.** FBS seems to accelerate aggregates growth, particularly during the first hours in culture. Scale bars, 50µm.

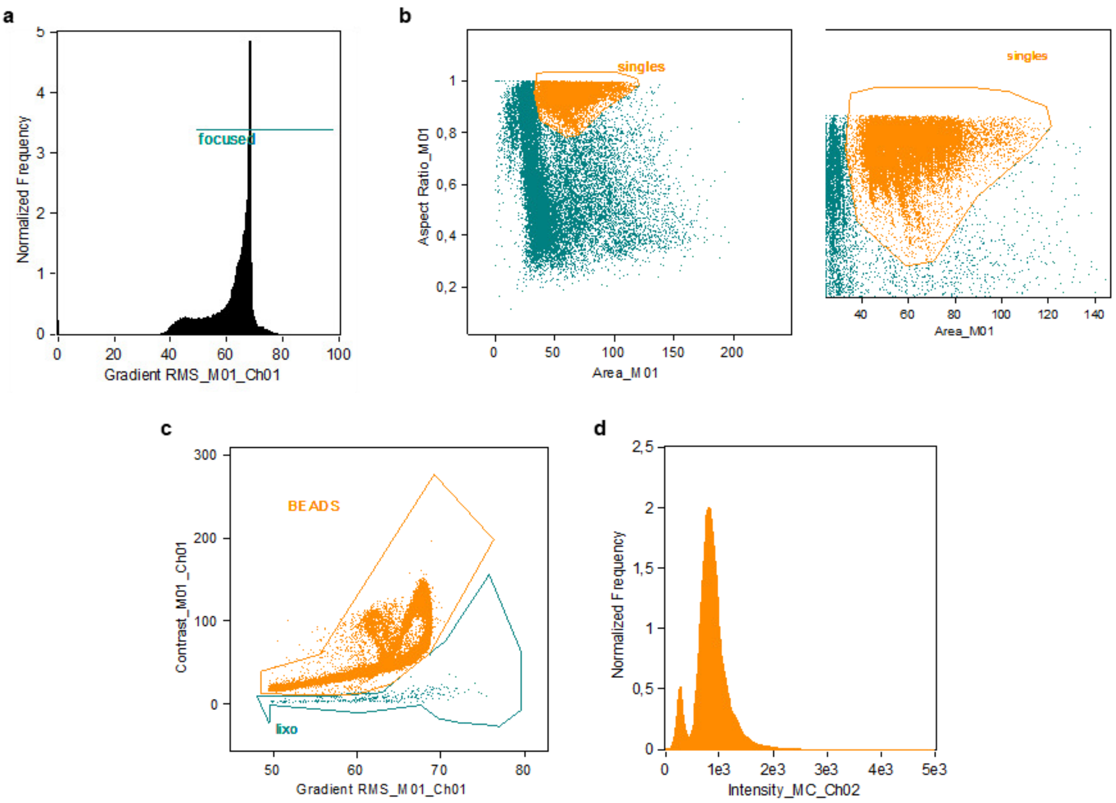

Supplementary Figure 2. Step-wise imaging flow cytometry analysis of EVs-coupled to beads. (a) Focused beads were gated using a histogram of gradient RMS, which represents the Root Mean Square for image sharpness. **(b)** An aspect ratio/area scatter plot was used to select single beads based on their roundness (aspect ratio close to 1) and characteristic area (40-120 pixels). **(c)** A contrast/gradient RMS plot was used to distinguish beads from debris, which showed small changes of pixel values (contrast near 0). **(d)** The intensity of the fluorescence staining (Alexa Fluor 488) was plotted in a histogram of channel 2 intensity.

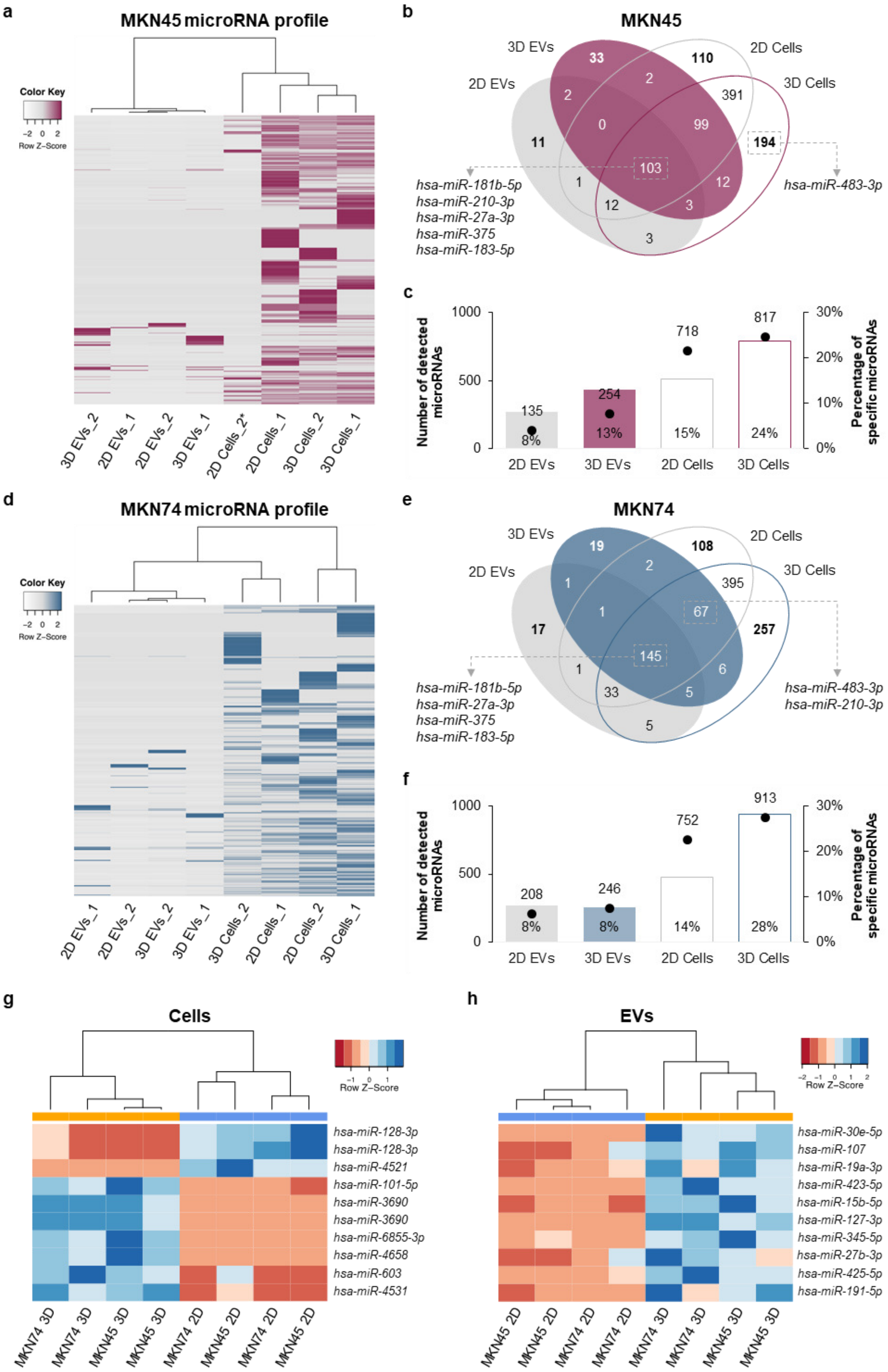
a
MKN45 microRNA profile
Color Key
Row Z-Score
3D EVs_2
2D EVs_1
2D EVs_2
3D EVs_1
2D Cells_2*
2D Cells_1
3D Cells_2
3D Cells_1
b
MKN45
3D EVs
2D Cells
2D EVs
3D Cells
hsa-miR-181b-5p
hsa-miR-210-3p
hsa-miR-27a-3p
hsa-miR-375
hsa-miR-183-5p
hsa-miR-483-3p
c
Number of detected microRNAs
Percentage of specific microRNAs
2D EVs
3D EVs
2D Cells
3D Cells
d
MKN74 microRNA profile
Color Key
Row Z-Score
2D EVs_1
2D EVs_2
3D EVs_2
3D EVs_1
3D Cells_2
2D Cells_1
2D Cells_2
3D Cells_1
e
MKN74
3D EVs
2D Cells
2D EVs
3D Cells
hsa-miR-181b-5p
hsa-miR-27a-3p
hsa-miR-375
hsa-miR-183-5p
hsa-miR-483-3p
hsa-miR-210-3p
f
Number of detected microRNAs
Percentage of specific microRNAs
2D EVs
3D EVs
2D Cells
3D Cells
g
Cells
Row Z-Score
hsa-miR-128-3p
hsa-miR-128-3p
hsa-miR-4521
hsa-miR-101-5p
hsa-miR-3690
hsa-miR-3690
hsa-miR-6855-3p
hsa-miR-4658
hsa-miR-603
hsa-miR-4531
MKN74 3D
MKN74 3D
MKN45 3D
MKN45 3D
MKN74 2D
MKN45 2D
MKN74 2D
MKN45 2D
h
EVs
Row Z-Score
hsa-miR-30e-5p
hsa-miR-107
hsa-miR-19a-3p
hsa-miR-423-5p
hsa-miR-15b-5p
hsa-miR-127-3p
hsa-miR-345-5p
hsa-miR-27b-3p
hsa-miR-425-5p
hsa-miR-191-5p
MKN45 2D
MKN45 2D
MKN74 2D
MKN74 2D
MKN74 3D
MKN74 3D
MKN45 3D
MKN45 3D

Supplementary Figure 3. Individual analysis of microRNA repertoires of MKN45 (a-c) and MKN74 (d-f) cells and EVs. (a, d) Heatmap and dendrogram of microRNA profiles of GC EVs and cells in 2D and 3D cultures (Z-score normalized expression values). **(b, e)** Venn diagrams showing the distribution of detected microRNAs. **(c, f)** Plots showing the number of total microRNAs and percentage of specific microRNAs detected in each condition (2 biological replicates). **(g, h)** The top-10 microRNAs in cells **(g)** and EVs **(h)** identified by supervised hierarchical clustering according to un-paired t-tests, and subjected to transformation of the normalized expression intensities to z-scores.

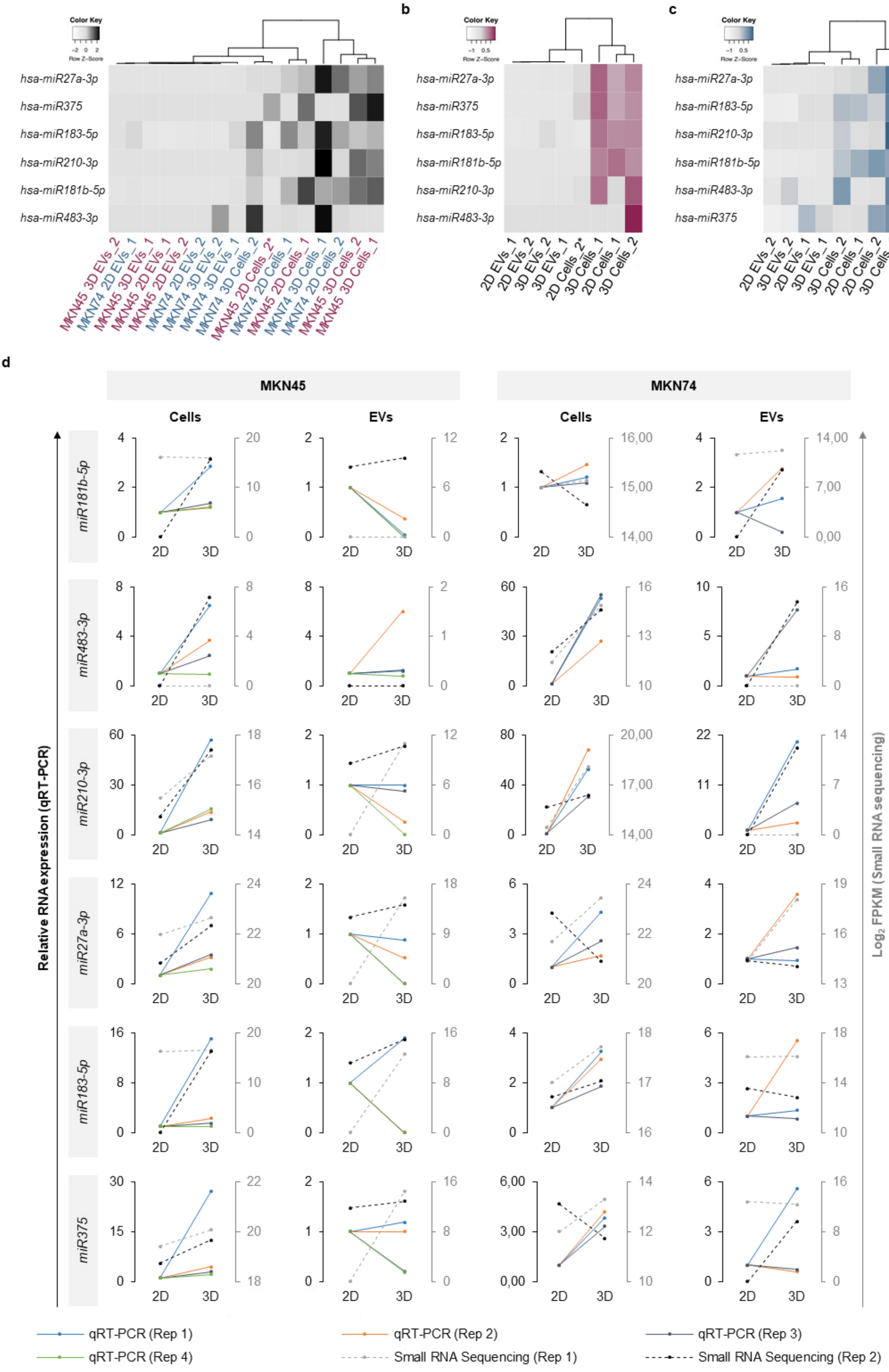

Color Key
Row Z-Score
hsa-miR27a-3p
hsa-miR375
hsa-miR183-5p
hsa-miR210-3p
hsa-miR181b-5p
hsa-miR483-3p
MKN45 3D EVs_2
MKN74 2D EVs_1
MKN45 3D EVs_1
MKN45 2D EVs_1
MKN45 2D EVs_2
MKN74 2D EVs_2
MKN74 3D EVs_2
MKN74 3D EVs_1
MKN74 3D Cells_2
MKN45 2D Cells_2*
MKN74 2D Cells_1
MKN45 2D Cells_1
MKN74 3D Cells_1
MKN74 2D Cells_2
MKN45 3D Cells_2
MKN45 3D Cells_1
b
2D EVs_1
2D EVs_2
3D EVs_2
3D EVs_1
2D Cells_2*
3D Cells_1
2D Cells_1
3D Cells_2
c
2D EVs_2
3D EVs_2
2D EVs_1
3D EVs_1
3D Cells_2
2D Cells_1
2D Cells_2
3D Cells_1
d
MKN45
MKN74
Cells
EVs
miR181b-5p
miR483-3p
miR210-3p
miR27a-3p
miR183-5p
miR375
2D
3D
Relative RNA expression (qRT-PCR)
Log2 FPKM (Small RNA sequencing)
qRT-PCR (Rep 1)
qRT-PCR (Rep 2)
qRT-PCR (Rep 3)
qRT-PCR (Rep 4)
Small RNA Sequencing (Rep 1)
Small RNA Sequencing (Rep 2)

Supplementary Figure 4. Validation of the small RNA sequencing data by quantitative real-time PCR. (a-c) Heatmap and dendrogram of six microRNAs detected in MKN45 and MKN74 EVs and cells in 2D and 3D cultures (Z-score normalized expression values). Analysis performed with data for both MKN45 and MKN74 GC cell lines **(a)**, and individually for MKN45 **(b)** or MKN74 **(c)**. **(d)** Quantitative RT-PCR and small RNA sequencing data of the six microRNAs from MKN45 and MKN74 cells and EVs selected for validation. Each biological replicate is independently represented.

Pathways (miRWalk)	hsa04115 p53 signaling pathway	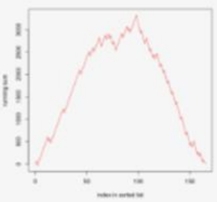	enriched	1.90629e-10	100	hsa-miR-181a-5p; hsa-miR-30b-5p; hsa-let-7b-5p; hsa-miR-23a-3p; hsa-miR-21-5p; hsa-miR-200c-3p; hsa-miR-27a-3p; hsa-miR-30d-5p; hsa-miR-34a-5p; hsa-miR-191-5p; hsa-miR-125a-5p; hsa-
Pathways (miRWalk)	WP382 MAPK signaling pathway	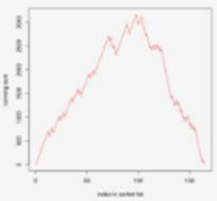	enriched	8.44947e-10	105	hsa-miR-181a-5p; hsa-miR-223-3p; hsa-miR-30b-5p; hsa-let-7b-5p; hsa-miR-23a-3p; hsa-miR-21-5p; hsa-miR-200c-3p; hsa-miR-27a-3p; hsa-miR-30d-5p; hsa-miR-34a-5p; hsa-miR-191-5p; hsa-miR-
Pathways (miRWalk)	WP366 TGF beta Signaling Pathway1	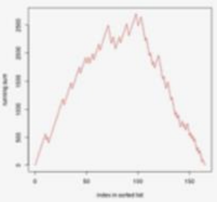	enriched	1.78309e-07	108	hsa-miR-181a-5p; hsa-miR-223-3p; hsa-miR-30b-5p; hsa-let-7b-5p; hsa-miR-23a-3p; hsa-miR-21-5p; hsa-miR-200c-3p; hsa-miR-27a-3p; hsa-miR-30d-5p; hsa-miR-34a-5p; hsa-miR-125a-5p; hsa-
Pathways (miRWalk)	P04393 Ras Pathway	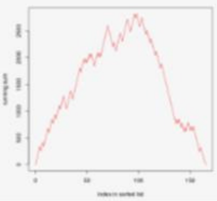	enriched	2.51788e-07	80	hsa-miR-181a-5p; hsa-miR-223-3p; hsa-miR-30b-5p; hsa-let-7b-5p; hsa-miR-21-5p; hsa-miR-200c-3p; hsa-miR-30d-5p; hsa-miR-34a-5p; hsa-miR-191-5p; hsa-miR-125a-5p; hsa-miR-221-3p; hsa-miR- [illegible]

Supplementary Figure 5. GSEA analysis results. GSEA analysis of 164 microRNAs detected in EVs from 3D culture, showing pathways highly enriched in 3D EVs.

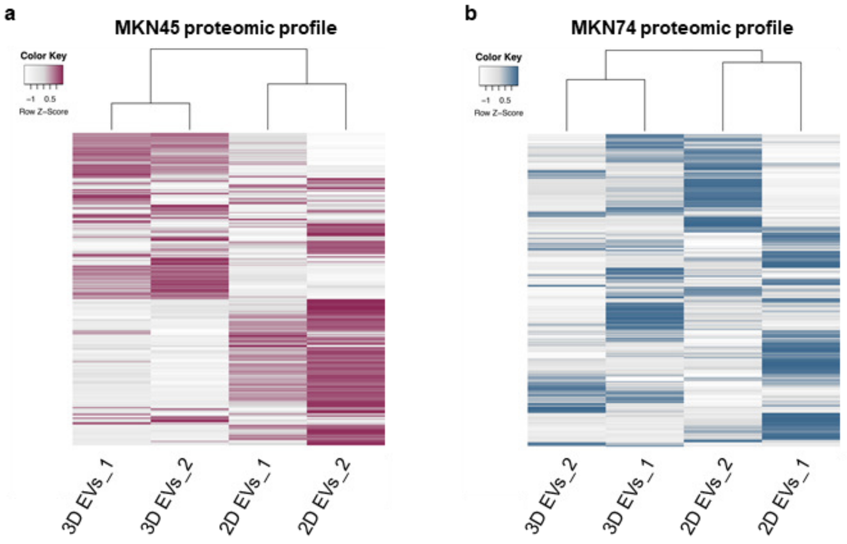

Supplementary Figure 6. Protein expression profiles of EVs isolated from 2D and 3D cultures. Heatmap and dendrogram of proteins detected individually in MKN45 **(a)** and MKN74 **(b)** EVs isolated from 2D and 3D cultures (Z-score normalized expression values).

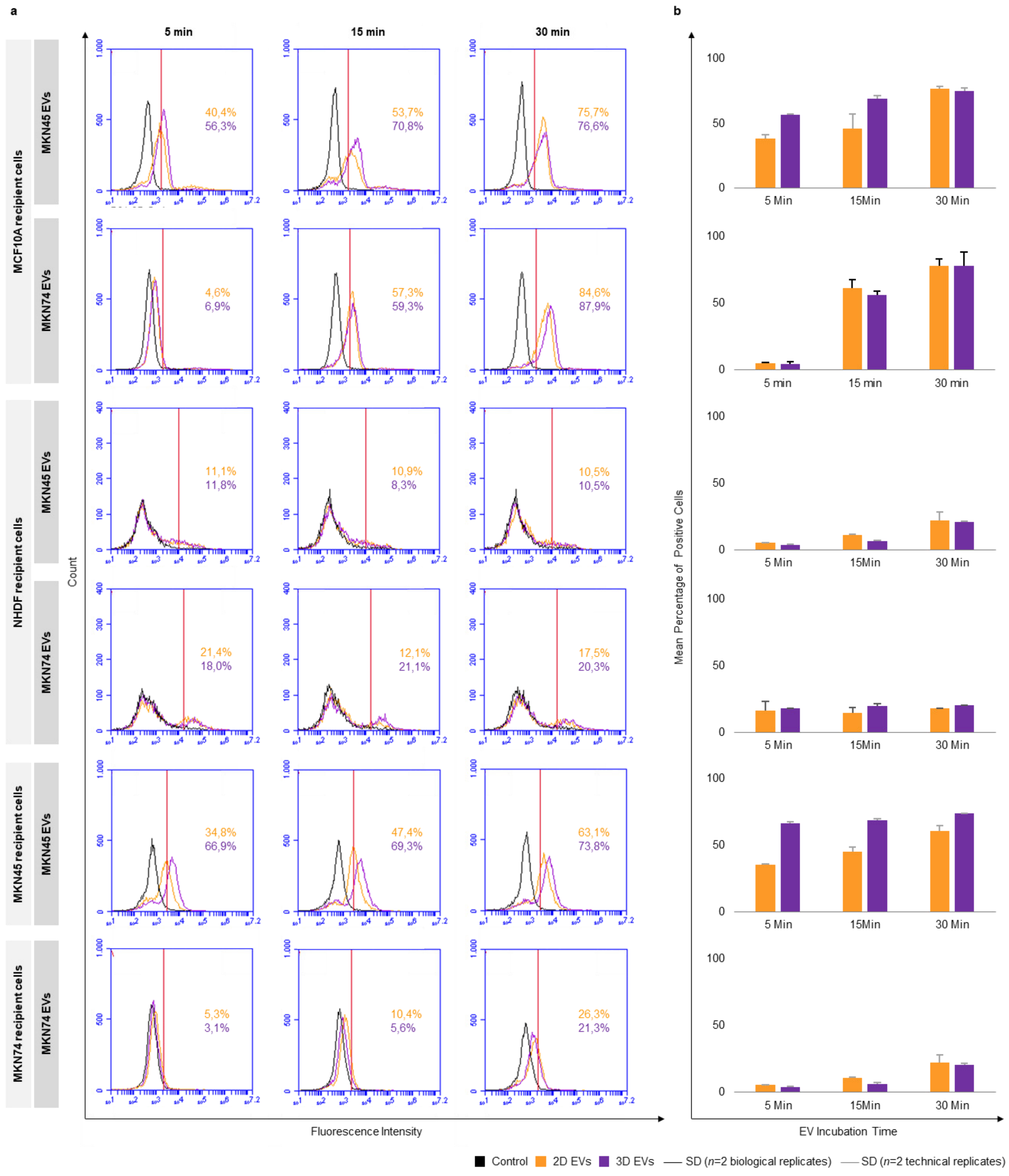

Supplementary Figure 7. Association between cells and EVs isolated from 2D and 3D cultures. Representative histograms showing the fluorescence intensity of EVs associated with different recipient cells (MCF10A, NHDF, MKN45 and MKN74 cells). Black curves for the control, orange curves for association with 2D EVs and purple for association with 3D EVs **(a).** Graphs showing the mean percentage of positive cells, i.e. cells that are associated with fluorescently labelled EVs **(b).**

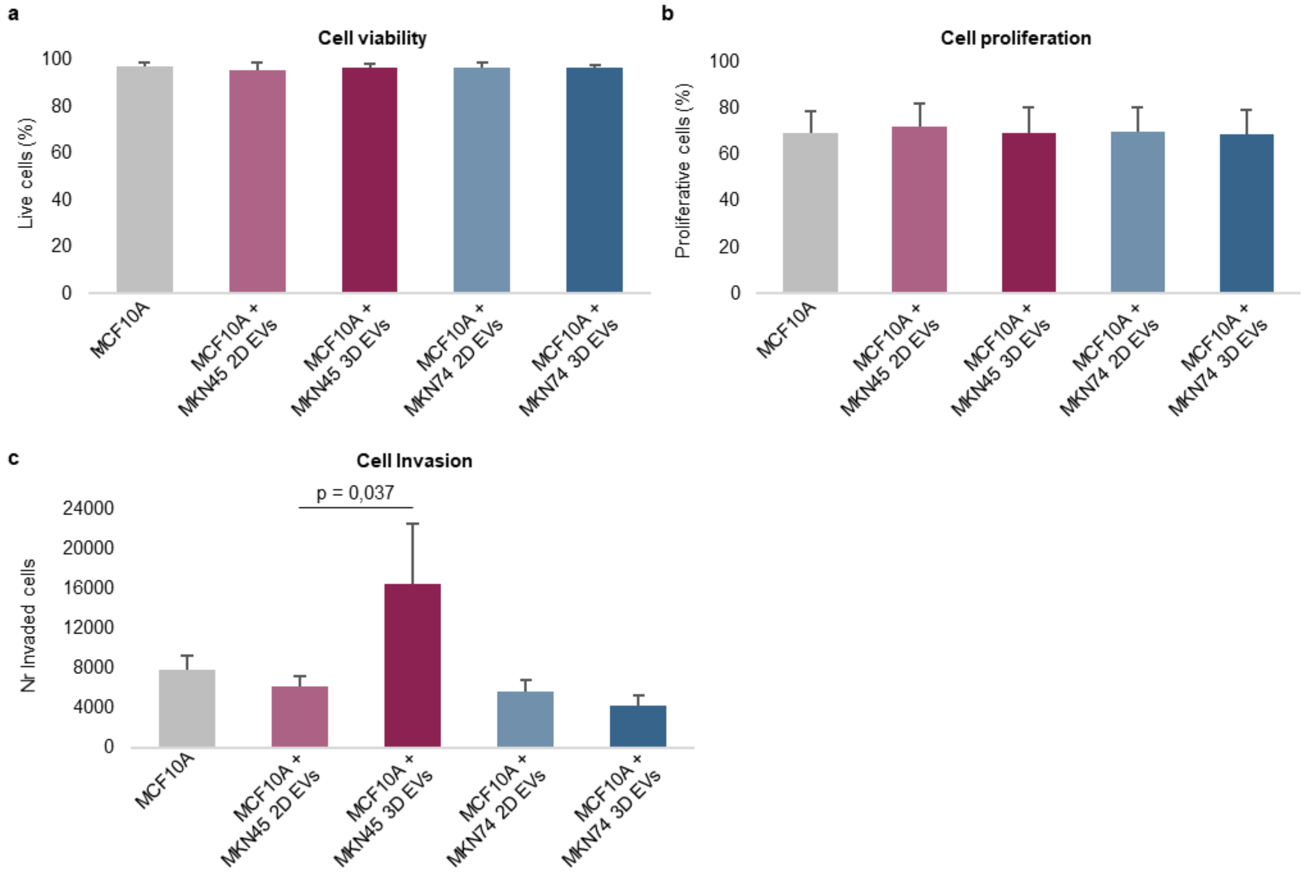

Supplementary Figure 8. Functional impact of EVs isolated from 2D and 3D cultures. Graphs showing cell viability (A), proliferation (B) and invasion (C) of MCF10A cells untreated and treated with EVs derived from 2D and 3D cultures of MKN45 and MKN74 cells. Results represent the mean ± standard deviation of three biological replicates. Data was analyzed with one-way analysis of variance (ANOVA) with Tukey's multiple comparisons test.

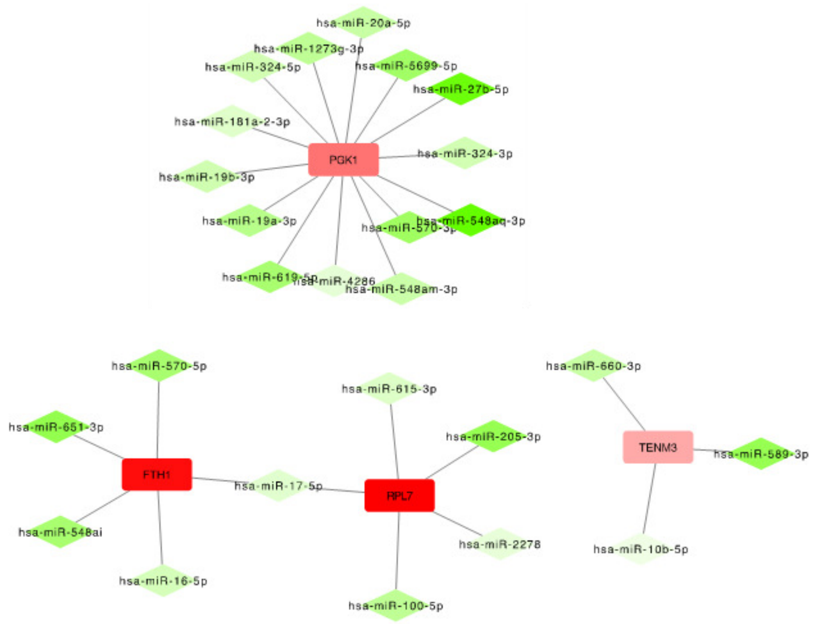

Supplementary Figure 9. Protein set enrichment and integrative network analysis. Three representative clusters showing that microRNAs (rhombs) target specific proteins (rectangles), being de-regulated in opposite direction. All proteins in EVs are down-regulated (red) while the microRNAs targeting these proteins are up-regulated (green).

Supplementary Tables

Supplementary Table 1. Technical information regarding the 2D and 3D culture systems used in this study

		3D cultures	2D cultures
**Working unit**		9.15 cm^2 agarose matrix	175 cm^2 flask
**Volume per working unit (mL)**		6	20
**Total period of culture (days)**		7	5
**Period of culture in EV-depleted medium (days)**		6	2
**Number of cells at time of collection per working unit**	**MKN45**	$2,70x10^6 \pm 6,69x10^5$	$1,77x10^7 \pm 8,36x10^6$
	**MKN74**	$2,06x10^6 \pm 1,16x10^6$	$1,88x10^7 \pm 6,15x10^6$
**Number of EVs collected per working unit**	**MKN45**	$2,42x10^9 \pm 1,09x10^9$	$8,49x10^9 \pm 1,91x10^9$
	**MKN74**	$2,02x10^9 \pm 1,35x10^9$	$6,09x10^9 \pm 1,87x10^9$
**Number of EVs collected per mL**	**MKN45**	$4,04x10^8 \pm 1,82x10^8$	$4,25x10^8 \pm 9,55x10^7$
	**MKN74**	$3,37x10^8 \pm 2,25x10^8$	$3,04x10^8 \pm 9,37x10^7$
**Number of EVs collected per cell**	**MKN45**	1019,59 ± 687,36	636,49 ± 374,97
	**MKN74**	1656,11 ± 1621,61	365,10 ± 165,67

Results represent the mean ± standard deviation of four biological replicates.

Supplementary Table 2. Total number of genes detected in each biological replicate according to specific small RNA classes

Small RNA Classes	Number of Annotated Genes	Total number of genes detected in each sample															
		MKN45								MKN74							
		Cells				EVs				Cells				EVs			
		2D		3D		2D		3D		2D		3D		2D		3D	
		Rep1	Rep2	Rep1	Rep2	Rep1	Rep2	Rep1	Rep2	Rep1	Rep2	Rep1	Rep2	Rep1	Rep2	Rep1	Rep2
**miRNA**	2803	778	162	687	758	27	148	212	217	660	711	843	813	168	177	184	219
**misc_RNA**	1100	112	9	165	166	64	98	87	102	159	84	166	407	53	71	22	87
**Mt_tRNA**	3	3	2	3	3	1	1	0	0	3	3	3	3	0	1	1	2
**ribozyme**	6	1	0	2	0	0	0	0	0	0	1	0	0	0	0	0	0
**rRNA**	510	253	88	230	297	11	18	34	31	316	184	196	419	20	42	11	48
**scaRNA**	9	7	2	8	9	0	2	1	1	8	6	7	9	0	1	0	1
**scRNA**	1	1	0	1	0	0	0	0	0	1	1	1	0	0	0	0	1
**snoRNA**	234	96	23	89	104	4	9	13	7	92	88	100	123	3	4	2	8
**snRNA**	1801	511	162	440	641	15	23	29	35	576	319	362	920	16	36	9	45
**Total**	6467	1762	448	1625	1978	122	299	376	393	1815	1397	1678	2694	260	332	229	411

Supplementary Table 3. Over-representation analysis of 10 microRNAs exclusively present in EVs from 3D culture

Category	Subcategory	Enrichment	p-value	expected	observed	miRNAs/precursors
**Diseases (miRWalk)**	Burkitt Lymphoma	over-represented	8,3173E-05	0,157895	3	hsa-miR-155-5p; hsa-miR-143-3p; hsa-miR-127-3p
**Organs (miRWalk)**	Germinal Center	over-represented	0,00052901	0,0560748	2	hsa-miR-155-5p; hsa-miR-127-3p
**Organs (miRWalk)**	B-Lymphocytes	over-represented	0,00342595	0,476636	3	hsa-miR-155-5p; hsa-miR-143-3p; hsa-miR-127-3p
**Target genes (miRTarbase)**	BCL6	over-represented	0,0045355	0,128571	2	hsa-miR-155-5p; hsa-miR-127-3p
**Target genes (miRTarbase)**	HK2	over-represented	0,0045355	0,128571	2	hsa-miR-155-5p; hsa-miR-143-3p
**Target genes (miRTarbase)**	MYO6	over-represented	0,0045355	0,128571	2	hsa-miR-155-5p; hsa-miR-143-3p
**Diseases (miRWalk)**	Lymphoma	over-represented	0,00553097	0,552632	3	hsa-miR-155-5p; hsa-miR-143-3p; hsa-miR-127-3p
**Gene Ontology (miRWalk)**	GO0042092 type 2 immune response	over-represented	0,00631842	0,15	2	hsa-miR-155-5p; hsa-miR-127-3p
**Target genes (miRTarbase)**	KRAS	over-represented	0,00838286	0,171429	2	hsa-miR-155-5p; hsa-miR-143-3p
**Gene Ontology (miRWalk)**	GO0002829 negative regulation of type 2 immune response	over-represented	0,0107243	0,192857	2	hsa-miR-155-5p; hsa-miR-127-3p
**Target genes (miRTarbase)**	FNDC3B	over-represented	0,0107243	0,192857	2	hsa-miR-155-5p; hsa-miR-143-3p
**Diseases (miRWalk)**	Lymphoma B-Cell	over-represented	0,0160689	0,236842	2	hsa-miR-155-5p; hsa-miR-143-3p
**Gene Ontology (miRWalk)**	GO0034968 histone lysine methylation	over-represented	0,0193664	0,257143	2	hsa-miR-155-5p; hsa-miR-127-3p
**Gene Ontology (miRWalk)**	GO0045579 positive regulation of b cell differentiation	over-represented	0,0193664	0,257143	2	hsa-miR-155-5p; hsa-miR-127-3p
**Gene Ontology (miRWalk)**	GO0004396 hexokinase activity	over-represented	0,0227713	0,278571	2	hsa-miR-155-5p; hsa-miR-143-3p
**Gene Ontology (miRWalk)**	GO0034405 response to fluid shear stress	over-represented	0,0227713	0,278571	2	hsa-miR-155-5p; hsa-miR-143-3p
**Gene Ontology (miRWalk)**	GO0042730 fibrinolysis	over-represented	0,0227713	0,278571	2	hsa-miR-155-5p; hsa-miR-143-3p
**Gene Ontology (miRWalk)**	GO0045334 clathrin coated endocytic vesicle	over-represented	0,0227713	0,278571	2	hsa-miR-155-5p; hsa-miR-143-3p
**Gene Ontology (miRWalk)**	GO0005536 glucose binding	over-represented	0,026431	0,3	2	hsa-miR-155-5p; hsa-miR-143-3p
**Gene Ontology (miRWalk)**	GO0030195 negative	over-represented	0,026431	0,3	2	hsa-miR-155-5p; hsa-miR-143-3p

	regulation of blood coagulation					
**Gene Ontology (miRWalk)**	GO0044027 hypermethylation of cpg island	over-represented	0,026431	0,3	2	hsa-miR-155-5p; hsa-miR-143-3p
**Gene Ontology (miRWalk)**	GO0051385 response to mineralocorticoid stimulus	over-represented	0,026431	0,3	2	hsa-miR-155-5p; hsa-miR-143-3p
**Gene Ontology (miRWalk)**	GO0050727 regulation of inflammatory response	over-represented	0,0275727	0,921429	3	hsa-miR-155-5p; hsa-miR-143-3p; hsa-miR-127-3p
**Gene Ontology (miRWalk)**	GO0000060 protein import into nucleus translocation	over-represented	0,0295902	0,942857	3	hsa-miR-155-5p; hsa-miR-143-3p; hsa-miR-127-3p
**Gene Ontology (miRWalk)**	GO0000302 response to reactive oxygen species	over-represented	0,0303409	0,321429	2	hsa-miR-155-5p; hsa-miR-143-3p
**Gene Ontology (miRWalk)**	GO0002634 regulation of germinal center formation	over-represented	0,0303409	0,321429	2	hsa-miR-155-5p; hsa-miR-127-3p
**Gene Ontology (miRWalk)**	GO0030275 lrr domain binding	over-represented	0,0303409	0,321429	2	hsa-miR-155-5p; hsa-miR-143-3p
**Gene Ontology (miRWalk)**	GO0046324 regulation of glucose import	over-represented	0,0303409	0,321429	2	hsa-miR-155-5p; hsa-miR-143-3p
**Gene Ontology (miRWalk)**	GO0061045 negative regulation of wound healing	over-represented	0,0303409	0,321429	2	hsa-miR-155-5p; hsa-miR-143-3p
**Gene Ontology (miRWalk)**	GO0002467 germinal center formation	over-represented	0,0344966	0,342857	2	hsa-miR-155-5p; hsa-miR-127-3p
**Gene Ontology (miRWalk)**	GO0010757 negative regulation of plasminogen activation	over-represented	0,0344966	0,342857	2	hsa-miR-155-5p; hsa-miR-143-3p
**Gene Ontology (miRWalk)**	GO0032287 peripheral nervous system myelin maintenance	over-represented	0,0344966	0,342857	2	hsa-miR-155-5p; hsa-miR-143-3p
**Gene Ontology (miRWalk)**	GO0035022 positive regulation of rac protein signal transduction	over-represented	0,0344966	0,342857	2	hsa-miR-155-5p; hsa-miR-143-3p
**Pathways (miRWalk)**	WP272 Blood Clotting Cascade	over-represented	0,0359716	0,350365	2	hsa-miR-155-5p; hsa-miR-143-3p
**Gene Ontology (miRWalk)**	GO0016461 unconventional myosin complex	over-represented	0,0388936	0,364286	2	hsa-miR-155-5p; hsa-miR-143-3p
**Gene Ontology (miRWalk)**	GO0051602 response to electrical stimulus	over-represented	0,0388936	0,364286	2	hsa-miR-155-5p; hsa-miR-127-3p
**Gene Ontology (miRWalk)**	GO0002903 negative regulation of b cell apoptotic	over-represented	0,0435274	0,385714	2	hsa-miR-155-5p; hsa-miR-127-3p

	process					
**Gene Ontology (miRWalk)**	GO0003886 dna cytosine 5 methyltransferase activity	over-represented	0,0435274	0,385714	2	hsa-miR-155-5p; hsa-miR-143-3p
**Gene Ontology (miRWalk)**	GO0006809 nitric oxide biosynthetic process	over-represented	0,0435274	0,385714	2	hsa-miR-155-5p; hsa-miR-143-3p
**Gene Ontology (miRWalk)**	GO0035024 negative regulation of rho protein signal transduction	over-represented	0,0435274	0,385714	2	hsa-miR-155-5p; hsa-miR-127-3p
**Gene Ontology (miRWalk)**	GO0050829 defense response to gram negative bacterium	over-represented	0,0435274	0,385714	2	hsa-miR-155-5p; hsa-miR-143-3p
**Gene Ontology (miRWalk)**	GO0051918 negative regulation of fibrinolysis	over-represented	0,0435274	0,385714	2	hsa-miR-155-5p; hsa-miR-143-3p
**Gene Ontology (miRWalk)**	GO0007281 germ cell development	over-represented	0,0437911	1,07143	3	hsa-miR-155-5p; hsa-miR-143-3p; hsa-miR-127-3p
**Gene Ontology (miRWalk)**	GO0030194 positive regulation of blood coagulation	over-represented	0,0483936	0,407143	2	hsa-miR-155-5p; hsa-miR-143-3p
**Gene Ontology (miRWalk)**	GO0032228 regulation of synaptic transmission gabaergic	over-represented	0,0483936	0,407143	2	hsa-miR-155-5p; hsa-miR-143-3p
**Gene Ontology (miRWalk)**	GO0032757 positive regulation of interleukin 8 production	over-represented	0,0483936	0,407143	2	hsa-miR-155-5p; hsa-miR-143-3p
**Gene Ontology (miRWalk)**	GO0043589 skin morphogenesis	over-represented	0,0483936	0,407143	2	hsa-miR-155-5p; hsa-miR-143-3p
**Gene Ontology (miRWalk)**	GO0051926 negative regulation of calcium ion transport	over-represented	0,0483936	0,407143	2	hsa-miR-155-5p; hsa-miR-143-3p

Supplementary Table 4. Over-representation analysis of 64 microRNAs detected in EVs from 3D culture, absent in EVs from 2D culture and present in both, cells growing in 2D and 3D cultures

Category	Subcategory	Enrichment	p-value	expected	observed	miRNAs/precursors
**Target genes (miRTarbase)**	PIP5K1A	over-represented	0,0100419	2,03571	5	hsa-miR-222-3p; hsa-miR-1226-3p; hsa-miR-132-3p; hsa-miR-615-3p; hsa-miR-744-5p
**Organs (miRWalk)**	Blood Platelets	over-represented	0,0128746	3,18692	7	hsa-let-7e-5p; hsa-miR-10a-5p; hsa-let-7d-3p; hsa-let-7c-5p; hsa-miR-151a-5p; hsa-let-7i-5p; hsa-miR-28-5p
**Target genes (miRTarbase)**	OTUD5	over-represented	0,0257678	1,62857	4	hsa-let-7e-5p; hsa-miR-339-5p; hsa-miR-1260b; hsa-miR-505-3p
**Target genes (miRTarbase)**	RPA1	over-represented	0,0257678	1,62857	4	hsa-let-7e-5p; hsa-miR-31-5p; hsa-miR-339-5p; hsa-miR-615-3p
**Gene Ontology (miRWalk)**	GO0048846 axon extension involved in axon guidance	over-represented	0,0409112	2,44286	5	hsa-let-7e-5p; hsa-miR-324-5p; hsa-miR-26b-5p; hsa-miR-326; hsa-miR-330-3p
**Target genes (miRTarbase)**	MCM2	over-represented	0,0409112	2,44286	5	hsa-miR-423-3p; hsa-miR-31-5p; hsa-miR-1226-3p; hsa-miR-615-3p; hsa-miR-500a-5p

Supplementary Table 5. GSEA Analysis of 164 microRNAs detected in EVs from 3D culture – top-20 hits

Category	Subcategory	Enrichment	p-value	observed	miRNAs/precursors
**Diseases (miRWalk)**	Neoplasms	enriched	4,03E-09	106	hsa-miR-181a-5p; hsa-miR-223-3p; hsa-let-7b-5p; hsa-miR-23a-3p; hsa-miR-21-5p; hsa-miR-200c-3p; hsa-miR-27a-3p; hsa-miR-34a-5p; hsa-miR-191-5p; hsa-miR-125a-5p; hsa-miR-221-3p; hsa-miR-451a; hsa-miR-93-5p; hsa-miR-205-5p; hsa-miR-192-5p; hsa-miR-320a; hsa-let-7f-5p; hsa-miR-20a-5p; hsa-miR-17-5p; hsa-miR-26a-5p; hsa-miR-193b-3p; hsa-let-7e-5p; hsa-miR-183-5p; hsa-miR-365a-3p; hsa-miR-197-3p; hsa-miR-19b-3p; hsa-miR-27b-3p; hsa-miR-122-5p; hsa-miR-24-3p; hsa-miR-103a-3p; hsa-miR-144-3p; hsa-miR-16-5p; hsa-miR-149-5p; hsa-miR-106b-5p; hsa-let-7g-5p; hsa-miR-200b-3p; hsa-miR-423-3p; hsa-miR-141-3p; hsa-miR-30c-5p; hsa-miR-23b-3p; hsa-miR-19a-3p; hsa-miR-195-5p; hsa-miR-203a-3p; hsa-miR-186-5p; hsa-miR-31-5p; hsa-miR-126-3p; hsa-let-7d-5p; hsa-miR-150-5p; hsa-miR-146a-5p; hsa-let-7a-5p; hsa-miR-18a-5p; hsa-miR-222-3p; hsa-miR-126-5p; hsa-miR-10a-5p; hsa-miR-200a-3p; hsa-miR-375; hsa-miR-145-5p; hsa-miR-324-5p; hsa-miR-185-5p; hsa-miR-133a-3p; hsa-miR-106a-5p; hsa-let-7d-3p; hsa-miR-135b-5p; hsa-miR-7-5p; hsa-miR-339-5p; hsa-miR-146b-5p; hsa-miR-378a-3p; hsa-miR-15a-5p; hsa-miR-125b-5p; hsa-miR-107; hsa-miR-25-3p; hsa-miR-429; hsa-miR-26b-5p; hsa-miR-92a-3p; hsa-miR-15b-5p; hsa-miR-29b-3p; hsa-miR-342-3p; hsa-miR-155-5p; hsa-miR-100-5p; hsa-miR-128-3p; hsa-miR-182-5p; hsa-miR-106b-3p; hsa-let-7c-5p; hsa-miR-345-5p; hsa-miR-29a-3p; hsa-miR-143-3p; hsa-miR-489-3p; hsa-miR-210-3p; hsa-miR-181b-5p; hsa-miR-127-3p; hsa-miR-151a-5p; hsa-miR-96-5p; hsa-miR-30a-3p; hsa-miR-326; hsa-miR-1226-3p; hsa-miR-199b-5p; hsa-miR-132-3p; hsa-miR-30c-1-3p; hsa-miR-330-3p; hsa-miR-124-3p; hsa-miR-199a-3p; hsa-miR-28-5p; hsa-miR-148b-3p; hsa-miR-193a-5p; hsa-miR-301a-3p; hsa-miR-22-3p
**Diseases (miRWalk)**	Carcinoma	enriched	9,14E-07	70	hsa-miR-181a-5p; hsa-miR-223-3p; hsa-let-7b-5p; hsa-miR-23a-3p; hsa-miR-21-5p; hsa-miR-200c-3p; hsa-miR-30d-5p; hsa-miR-34a-5p; hsa-miR-191-5p; hsa-miR-125a-5p; hsa-miR-221-3p; hsa-miR-451a; hsa-miR-93-5p; hsa-miR-205-5p; hsa-miR-192-5p; hsa-let-7f-5p; hsa-miR-17-5p; hsa-miR-26a-5p; hsa-miR-193b-3p; hsa-miR-183-5p; hsa-miR-197-3p; hsa-miR-19b-3p; hsa-miR-122-5p; hsa-miR-24-3p; hsa-miR-16-5p; hsa-miR-106b-5p; hsa-let-7g-5p; hsa-miR-200b-3p; hsa-miR-141-3p; hsa-miR-23b-3p; hsa-miR-19a-3p; hsa-miR-195-5p; hsa-miR-203a-3p; hsa-miR-186-5p; hsa-miR-31-5p; hsa-miR-126-3p; hsa-miR-146a-5p; hsa-let-7a-5p; hsa-miR-18a-5p; hsa-miR-222-3p; hsa-miR-200a-3p; hsa-miR-375; hsa-miR-145-5p; hsa-miR-185-5p; hsa-miR-133a-3p; hsa-miR-135b-5p; hsa-miR-7-5p; hsa-miR-146b-5p; hsa-miR-15a-5p; hsa-miR-125b-5p; hsa-miR-107; hsa-miR-429; hsa-miR-26b-5p; hsa-miR-29b-3p; hsa-miR-100-5p; hsa-miR-182-5p; hsa-let-7c-5p; hsa-miR-29a-3p; hsa-miR-143-3p; hsa-miR-489-3p; hsa-miR-210-3p; hsa-miR-181b-5p; hsa-miR-151a-5p; hsa-miR-1226-3p; hsa-miR-199b-5p; hsa-miR-124-3p; hsa-miR-199a-3p; hsa-miR-193a-5p; hsa-miR-301a-3p; hsa-miR-22-3p
**Diseases (miRWalk)**	Lung Neoplasms	enriched	2,12E-05	28	hsa-let-7b-5p; hsa-miR-21-5p; hsa-miR-200c-3p; hsa-miR-221-3p; hsa-miR-451a; hsa-miR-93-5p; hsa-let-7f-5p; hsa-miR-20a-5p; hsa-let-7e-5p; hsa-miR-183-5p; hsa-miR-197-3p; hsa-miR-16-5p; hsa-let-7g-5p; hsa-miR-200b-3p; hsa-miR-30c-5p; hsa-miR-19a-3p; hsa-miR-31-5p; hsa-miR-126-3p; hsa-let-7a-5p; hsa-miR-222-3p; hsa-miR-145-5p; hsa-miR-185-5p; hsa-miR-15a-5p; hsa-miR-107; hsa-miR-429; hsa-miR-29b-3p; hsa-miR-29a-3p; hsa-miR-22-3p
**Diseases (miRWalk)**	Neoplasm Metastasis	enriched	2,43E-04	49	hsa-miR-223-3p; hsa-miR-23a-3p; hsa-miR-21-5p; hsa-miR-200c-3p; hsa-miR-30d-5p; hsa-miR-34a-5p; hsa-miR-125a-5p; hsa-miR-451a; hsa-miR-205-5p; hsa-miR-320a; hsa-miR-20a-5p; hsa-miR-17-5p; hsa-miR-26a-5p; hsa-miR-193b-3p; hsa-miR-183-5p; hsa-miR-197-3p; hsa-miR-122-5p; hsa-miR-103a-3p; hsa-miR-144-3p; hsa-miR-16-5p; hsa-miR-200b-3p; hsa-miR-141-3p; hsa-miR-23b-3p; hsa-miR-31-5p; hsa-miR-150-5p; hsa-miR-146a-5p; hsa-let-7a-5p; hsa-miR-222-3p; hsa-miR-10a-5p; hsa-miR-200a-3p; hsa-miR-145-5p; hsa-miR-324-5p; hsa-miR-339-5p; hsa-miR-125b-5p; hsa-miR-107; hsa-miR-429; hsa-miR-26b-5p; hsa-miR-29b-3p; hsa-miR-155-5p; hsa-miR-182-5p; hsa-miR-194-5p; hsa-miR-486-5p; hsa-miR-29a-3p; hsa-miR-143-3p; hsa-miR-210-3p; hsa-miR-151a-5p; hsa-miR-326; hsa-miR-199b-5p; hsa-miR-124-3p
**Diseases (miRWalk)**	Inflammation	enriched	2,44E-04	30	hsa-miR-181a-5p; hsa-miR-223-3p; hsa-miR-21-5p; hsa-miR-221-3p; hsa-miR-192-5p; hsa-let-7f-5p; hsa-miR-19b-3p; hsa-miR-27b-3p; hsa-let-7g-5p; hsa-miR-141-3p; hsa-miR-19a-3p; hsa-miR-195-5p; hsa-miR-

					203a-3p; hsa-miR-31-5p; hsa-miR-126-3p; hsa-let-7d-5p; hsa-miR-146a-5p; hsa-let-7a-5p; hsa-miR-10a-5p; hsa-miR-200a-3p; hsa-miR-769-5p; hsa-miR-7-5p; hsa-miR-146b-5p; hsa-miR-107; hsa-miR-25-3p; hsa-miR-29b-3p; hsa-miR-155-5p; hsa-miR-181b-5p; hsa-let-7i-5p; hsa-miR-22-3p
**Diseases (miRWalk)**	Shock	enriched	4,49E-04	2	hsa-miR-223-3p; hsa-let-7b-5p
**Diseases (miRWalk)**	Carcinoma Hepatocellular	enriched	5,49E-04	43	hsa-miR-181a-5p; hsa-miR-223-3p; hsa-let-7b-5p; hsa-miR-23a-3p; hsa-miR-21-5p; hsa-miR-30d-5p; hsa-miR-191-5p; hsa-miR-125a-5p; hsa-miR-221-3p; hsa-miR-93-5p; hsa-miR-26a-5p; hsa-miR-193b-3p; hsa-miR-183-5p; hsa-miR-122-5p; hsa-miR-144-3p; hsa-miR-16-5p; hsa-miR-106b-5p; hsa-let-7g-5p; hsa-miR-23b-3p; hsa-miR-195-5p; hsa-miR-203a-3p; hsa-miR-186-5p; hsa-let-7a-5p; hsa-miR-18a-5p; hsa-miR-222-3p; hsa-miR-200a-3p; hsa-miR-375; hsa-miR-145-5p; hsa-miR-185-5p; hsa-miR-125b-5p; hsa-miR-29b-3p; hsa-let-7c-5p; hsa-miR-29a-3p; hsa-miR-143-3p; hsa-miR-210-3p; hsa-miR-181b-5p; hsa-miR-151a-5p; hsa-miR-199b-5p; hsa-miR-30c-1-3p; hsa-miR-124-3p; hsa-miR-199a-3p; hsa-miR-301a-3p; hsa-miR-22-3p
**Diseases (miRWalk)**	Breast Neoplasms	enriched	9,60E-04	48	hsa-miR-21-5p; hsa-miR-200c-3p; hsa-miR-27a-3p; hsa-miR-34a-5p; hsa-miR-125a-5p; hsa-miR-221-3p; hsa-miR-451a; hsa-miR-205-5p; hsa-let-7f-5p; hsa-miR-20a-5p; hsa-miR-17-5p; hsa-miR-193b-3p; hsa-miR-103a-3p; hsa-miR-16-5p; hsa-miR-200b-3p; hsa-miR-141-3p; hsa-miR-30c-5p; hsa-miR-203a-3p; hsa-miR-31-5p; hsa-miR-126-3p; hsa-miR-150-5p; hsa-miR-146a-5p; hsa-let-7a-5p; hsa-miR-222-3p; hsa-miR-200a-3p; hsa-miR-375; hsa-miR-145-5p; hsa-miR-185-5p; hsa-miR-7-5p; hsa-miR-339-5p; hsa-miR-146b-5p; hsa-miR-125b-5p; hsa-miR-107; hsa-miR-92a-3p; hsa-miR-29b-3p; hsa-miR-342-3p; hsa-miR-155-5p; hsa-miR-128-3p; hsa-miR-182-5p; hsa-miR-345-5p; hsa-miR-29a-3p; hsa-miR-181b-5p; hsa-miR-96-5p; hsa-miR-326; hsa-miR-1226-3p; hsa-miR-330-3p; hsa-miR-199a-3p; hsa-miR-22-3p
**Diseases (miRWalk)**	Insulin Resistance	enriched	1,12E-03	2	hsa-miR-181a-5p; hsa-miR-21-5p
**Diseases (miRWalk)**	Prostatic Neoplasms	enriched	1,13E-03	21	hsa-miR-21-5p; hsa-miR-200c-3p; hsa-miR-34a-5p; hsa-miR-221-3p; hsa-miR-205-5p; hsa-miR-26a-5p; hsa-miR-122-5p; hsa-miR-16-5p; hsa-miR-106b-5p; hsa-miR-141-3p; hsa-miR-126-3p; hsa-miR-146a-5p; hsa-let-7a-5p; hsa-miR-126-5p; hsa-miR-145-5p; hsa-miR-185-5p; hsa-miR-125b-5p; hsa-miR-107; hsa-miR-29b-3p; hsa-miR-143-3p; hsa-miR-330-3p
**Diseases (miRWalk)**	Colonic Neoplasms	enriched	1,21E-03	17	hsa-let-7b-5p; hsa-miR-21-5p; hsa-miR-34a-5p; hsa-miR-192-5p; hsa-miR-320a; hsa-miR-183-5p; hsa-miR-365a-3p; hsa-miR-106b-5p; hsa-miR-195-5p; hsa-miR-186-5p; hsa-miR-126-3p; hsa-let-7a-5p; hsa-miR-145-5p; hsa-miR-107; hsa-miR-25-3p; hsa-miR-15b-5p; hsa-miR-143-3p
**Diseases (miRWalk)**	Recurrence	enriched	1,45E-03	14	hsa-miR-21-5p; hsa-miR-191-5p; hsa-miR-125a-5p; hsa-miR-221-3p; hsa-miR-451a; hsa-miR-20a-5p; hsa-miR-26a-5p; hsa-miR-183-5p; hsa-miR-122-5p; hsa-miR-203a-3p; hsa-miR-29b-3p; hsa-miR-100-5p; hsa-miR-326; hsa-miR-124-3p
**Diseases (miRWalk)**	Glioblastoma	enriched	1,45E-03	24	hsa-miR-181a-5p; hsa-miR-21-5p; hsa-miR-34a-5p; hsa-miR-221-3p; hsa-miR-451a; hsa-miR-93-5p; hsa-miR-192-5p; hsa-miR-20a-5p; hsa-miR-17-5p; hsa-miR-26a-5p; hsa-miR-24-3p; hsa-miR-19a-3p; hsa-miR-195-5p; hsa-let-7d-5p; hsa-let-7a-5p; hsa-miR-18a-5p; hsa-miR-222-3p; hsa-miR-7-5p; hsa-miR-146b-5p; hsa-miR-92a-3p; hsa-miR-128-3p; hsa-miR-326; hsa-miR-199b-5p; hsa-miR-124-3p
**Diseases (miRWalk)**	Carcinoma Non-Small-Cell Lung	enriched	1,75E-03	11	hsa-miR-21-5p; hsa-miR-221-3p; hsa-miR-451a; hsa-miR-16-5p; hsa-let-7g-5p; hsa-miR-31-5p; hsa-miR-222-3p; hsa-miR-145-5p; hsa-miR-185-5p; hsa-miR-15a-5p; hsa-miR-107
**Diseases (miRWalk)**	Lymphoma	enriched	2,96E-03	21	hsa-miR-181a-5p; hsa-miR-30b-5p; hsa-let-7b-5p; hsa-miR-21-5p; hsa-miR-200c-3p; hsa-miR-34a-5p; hsa-let-7f-5p; hsa-miR-20a-5p; hsa-miR-17-5p; hsa-miR-26a-5p; hsa-miR-16-5p; hsa-miR-200b-3p; hsa-let-7a-5p; hsa-miR-200a-3p; hsa-miR-375; hsa-miR-15a-5p; hsa-miR-29b-3p; hsa-miR-155-5p; hsa-miR-29a-3p; hsa-miR-143-3p; hsa-miR-127-3p
**Diseases (miRWalk)**	Nevus	enriched	3,19E-03	3	hsa-let-7b-5p; hsa-miR-205-5p; hsa-miR-193b-3p
**Diseases (miRWalk)**	Encephalitis	enriched	4,78E-03	5	hsa-let-7b-5p; hsa-miR-21-5p; hsa-miR-34a-5p; hsa-miR-26a-5p; hsa-miR-145-5p
**Diseases (miRWalk)**	Cholangiocarcinoma	enriched	5,15E-03	4	hsa-miR-21-5p; hsa-miR-320a; hsa-miR-200b-3p; hsa-miR-141-3p
**Diseases (miRWalk)**	Stomach Neoplasms	enriched	5,90E-03	27	hsa-miR-223-3p; hsa-miR-23a-3p; hsa-miR-21-5p; hsa-miR-27a-3p; hsa-miR-34a-5p; hsa-miR-125a-5p; hsa-miR-221-3p; hsa-miR-451a; hsa-miR-93-5p; hsa-miR-16-5p; hsa-miR-106b-5p; hsa-miR-126-3p; hsa-

					miR-150-5p; hsa-miR-146a-5p; hsa-let-7a-5p; hsa-miR-222-3p; hsa-miR-375; hsa-miR-106a-5p; hsa-miR-107; hsa-miR-25-3p; hsa-miR-15b-5p; hsa-miR-29b-3p; hsa-miR-182-5p; hsa-miR-106b-3p; hsa-miR-143-3p; hsa-miR-148b-3p; hsa-miR-22-3p
**Diseases (miRWalk)**	Leukemia	enriched	5,92E-03	37	hsa-miR-181a-5p; hsa-miR-223-3p; hsa-miR-21-5p; hsa-miR-34a-5p; hsa-miR-125a-5p; hsa-miR-221-3p; hsa-miR-93-5p; hsa-miR-320a; hsa-miR-26a-5p; hsa-miR-19b-3p; hsa-miR-122-5p; hsa-miR-24-3p; hsa-miR-16-5p; hsa-miR-106b-5p; hsa-miR-126-3p; hsa-miR-150-5p; hsa-let-7a-5p; hsa-miR-222-3p; hsa-miR-10a-5p; hsa-miR-200a-3p; hsa-miR-145-5p; hsa-miR-106a-5p; hsa-miR-135b-5p; hsa-miR-15a-5p; hsa-miR-125b-5p; hsa-miR-107; hsa-miR-92a-3p; hsa-miR-29b-3p; hsa-miR-155-5p; hsa-miR-128-3p; hsa-miR-29a-3p; hsa-miR-505-3p; hsa-miR-143-3p; hsa-miR-181b-5p; hsa-miR-326; hsa-miR-124-3p; hsa-miR-22-3p

Supplementary Table 6. Top-20 proteins mostly regulated in EVs from 3D versus 2D culture conditions

Spalte1	median group 1	median group 2	stdv group 1	stdv group 2	fold change	log(2) fold change	t-Test raw p-Value	t-Test adjusted p-Value
**TSPAN14**	66,2	134,35	15,79	17,03	0,49	-1,02	0,0010822	0,29319
**ADGRG1**	11,9	171,55	27,3	38,33	0,07	-3,85	0,0013449	0,29319
**PTGFRN**	48,75	137,15	13,15	31,02	0,36	-1,49	0,0046935	0,47446
**EPHA2**	52,4	146,9	4,1	26,29	0,36	-1,49	0,0055448	0,47446
**CD151**	48,15	147,25	17,95	39,14	0,33	-1,61	0,0079419	0,47446
**PGK1**	129,8	82	18,22	8,38	1,58	0,66	0,0083176	0,47446
**CD46**	79,4	129,45	21,15	17,6	0,61	-0,71	0,0096387	0,47446
**ITGA3**	48,25	151,8	6,93	35,51	0,32	-1,65	0,010731	0,47446
**KPNB1**	82,9	113,05	12,28	10,8	0,73	-0,45	0,010915	0,47446
**ITGB1**	57,95	152,25	12,26	33,39	0,38	-1,39	0,011497	0,47446
**ADAM10**	85	117,95	13,25	16,62	0,72	-0,47	0,01197	0,47446
**DLG1**	57,3	136,55	13,95	39,74	0,42	-1,25	0,017437	0,60867
**EML2**	124	86,75	24,08	18,62	1,43	0,52	0,020416	0,60867
**ITGA2**	44,1	155,95	10,68	47,96	0,28	-1,82	0,020496	0,60867
**ANXA7**	76,3	125,35	12,07	19,29	0,61	-0,72	0,021508	0,60867
**RPL7**	131,7	62,2	43,47	16,28	2,12	1,08	0,027731	0,60867
**F11R**	62,8	125,85	7,93	37,48	0,5	-1	0,028809	0,60867
**ITGB5**	42,7	134,95	19,39	47,93	0,32	-1,66	0,030499	0,60867
**LAMP1**	59,75	120,7	15,69	42,41	0,5	-1,01	0,030733	0,60867
**ATP1A1**	61,65	143,6	6,16	37,51	0,43	-1,22	0,030785	0,60867

Supplementary Table 7. Detailed description of antibodies and TaqMan Advanced microRNA assays

Antibodies				
**ID**	**Clone**	**Supplier**	**Dilution**	**Application**
Integrin α6	3750	Cell Signaling Technology	1:800	WB
Integrin β1	D6S1W	Cell Signaling Technology	1:200	WB
EPHA-2	D4A2	Cell Signaling Technology	1:800	WB
GAPDH	G-9	Santa Cruz	1:500	WB
E-cadherin	C36	BD Biosciences	1:500	WB
p120 Catenin	H-90	BD Biosciences	1:500	WB
β-catenin	E-5	Santa Cruz	1:100	WB
Importin-β1	E1F1G	Cell Signaling Technology	1:200	WB
Tubulin	DLN-15303	Dianova	1:500	WB
LAMP1	D4O1S	Cell Signaling Technology	1:800	WB
JAM-A	1H2A9	Santa Cruz	1:300	WB
Integrin α3	C-18	Santa Cruz	1:400	WB
CD151	17327	Cell Signaling Technology	1:500	WB
Integrin α2	AF1740	R&D Systems	1:200	WB
Ki-67	SP6	ThermoFisher Scientific	1:200	IHC
E-cadherin	24E10	Cell Signaling Technology	1:200	ICC & IHC
Mucin -1		collaborators		ICC & IHC
CD9	ALB6	Santa Cruz	3:20	IFC
CD81	5A6	Santa Cruz	3:20	IFC
Flotillin-1	H-104	Santa Cruz	3:20	IFC
Cytochrome c	7H8	Santa Cruz	3:20	IFC

TaqMan Advanced microRNA assays		
**Assay name**	**Assay ID**	**Supplier**
hsa-miR-210-3p	477970_mir	ThermoFisher Scientific
hsa-miR-27a-3p	478384_mir	ThermoFisher Scientific
hsa-miR-183-5p	477937_mir	ThermoFisher Scientific
hsa-miR-375	478074_mir	ThermoFisher Scientific
hsa-miR-181b-5p	478583_mir	ThermoFisher Scientific
hsa-miR-483-3p	478122_mir	ThermoFisher Scientific
hsa-miR-30d-5p	478606_mir	ThermoFisher Scientific
hsa-miR-152-3p	477921_mir	ThermoFisher Scientific
hsa-miR-26b-5p	478418_mir	ThermoFisher Scientific
hsa-miR-155-5p	477927_mir	ThermoFisher Scientific

WB: Western Blotting; IHC: Immunohistochemistry; ICC: Immunocytochemistry; IFC: Imaging Flow Cytometry
